# High-frequency, precise modification of the tomato genome

**DOI:** 10.1186/s13059-015-0796-9

**Published:** 2015-11-06

**Authors:** Tomáš Čermák, Nicholas J. Baltes, Radim Čegan, Yong Zhang, Daniel F. Voytas

**Affiliations:** Department of Genetics, Cell Biology & Development and Center for Genome Engineering, University of Minnesota, Minneapolis, Minnesota 55455 USA; Department of Plant Developmental Genetics, Institute of Biophysics, Academy of Sciences of the Czech Republic, v.v.i., Královopolská 135, CZ-612 65, Brno, Czech Republic; Department of Biotechnology, School of Life Sciences and Technology, University of Electronic Science and Technology of China, 216 Main Building No. 4, Section 2, North Jianshe Road, Chengdu, 610054 P.R. China

## Abstract

**Background:**

The use of homologous recombination to precisely modify plant genomes has been challenging, due to the lack of efficient methods for delivering DNA repair templates to plant cells. Even with the advent of sequence-specific nucleases, which stimulate homologous recombination at predefined genomic sites by creating targeted DNA double-strand breaks, there are only a handful of studies that report precise editing of endogenous genes in crop plants. More efficient methods are needed to modify plant genomes through homologous recombination, ideally without randomly integrating foreign DNA.

**Results:**

Here, we use geminivirus replicons to create heritable modifications to the tomato genome at frequencies tenfold higher than traditional methods of DNA delivery (i.e., *Agrobacterium*). A strong promoter was inserted upstream of a gene controlling anthocyanin biosynthesis, resulting in overexpression and ectopic accumulation of pigments in tomato tissues. More than two-thirds of the insertions were precise, and had no unanticipated sequence modifications. Both TALENs and CRISPR/Cas9 achieved gene targeting at similar efficiencies. Further, the targeted modification was transmitted to progeny in a Mendelian fashion. Even though donor molecules were replicated in the vectors, no evidence was found of persistent extra-chromosomal replicons or off-target integration of T-DNA or replicon sequences.

**Conclusions:**

High-frequency, precise modification of the tomato genome was achieved using geminivirus replicons, suggesting that these vectors can overcome the efficiency barrier that has made gene targeting in plants challenging. This work provides a foundation for efficient genome editing of crop genomes without the random integration of foreign DNA.

**Electronic supplementary material:**

The online version of this article (doi:10.1186/s13059-015-0796-9) contains supplementary material, which is available to authorized users.

## Background

**Fig. 1:**
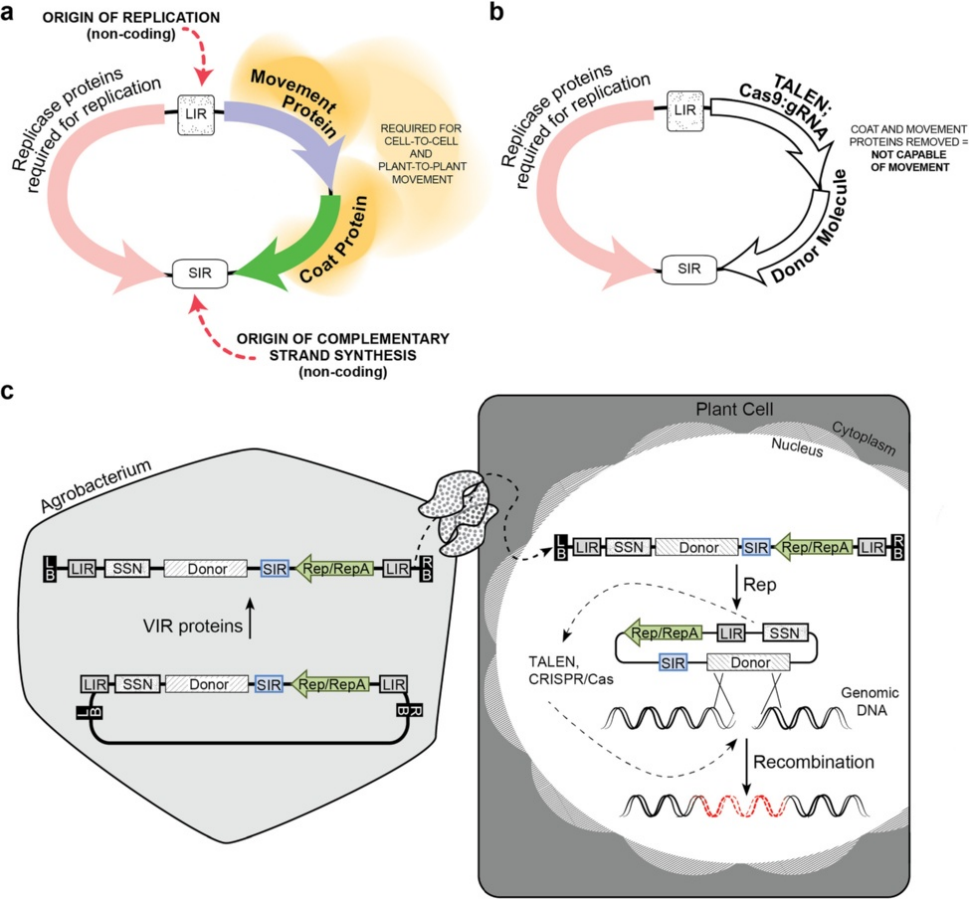
Gene targeting with geminivirus replicons. **a** Structure of the bean yellow dwarf virus (BeYDV) genome. The single-stranded DNA genome encodes three major functions: replicase proteins (Rep and RepA) mediate rolling circle replication, and movement and coat proteins are essential for viral movement. The long intergenic region (*LIR*) is the origin of replication and also functions as a bidirectional promoter that drives expression of viral genes. The short intergenic region (*SIR*) is the origin of C-strand synthesis and contains transcription termination and polyadenylation signals. **b** Structure of BeYDV genome modified for gene targeting. Coding sequences for movement and coat proteins were replaced with the site-specific nuclease and donor template for gene targeting. The modified virus is not capable of infection due to the lack of essential viral proteins. Further, the size exceeds the limit for successful packaging and cell-to-cell movement. The replication function is preserved, and the vector can replicate when delivered to plant cells by transformation. **c** Illustration of gene targeting with the modified BeYDV vector through *Agrobacterium*-mediated transformation. The BeYDV genome, containing the nuclease and donor template for gene targeting, is cloned into a transfer DNA (T-DNA) vector. One LIR is placed on each side of the viral genome to ensure release from the T-DNA in the plant cell. During *Agrobacterium* infection, linear T-DNA molecules are delivered to the nucleus of a plant cell, where the viral genome is replicationally released in a circular form and amplified into thousands of copies by rolling circle replication, mediated by the replicase proteins expressed from the LIR. The nuclease expressed from the viral genome induces DSBs at the target locus, and the donor template is copied into the target site by homology-directed repair. The high copy number of donor templates increases the frequency of gene targeting. *LB* left T-DNA border, *SSN* sequence-specific nuclease, *RB* right T-DNA border

The ability to precisely edit genomes holds much promise for advancing both basic and applied plant research. Already in many plant species, loss of function mutations can be created using sequence-specific nucleases that introduce double-strand breaks (DSBs) in coding sequences [1]. Mutagenesis results when the broken chromosomes are repaired imprecisely through non-homologous end joining (NHEJ), and small insertions/deletions (indels) are created at the break site. Repair of DSBs through homologous recombination (HR), however, offers a much richer spectrum of possibilities for modifying plant genomes, ranging from introducing single nucleotide substitutions to the seamless integration of multiple transgenes at a target locus. HR-based repair, or gene targeting (GT), uses information from an exogenously supplied DNA donor template to repair the break, and information is copied from the donor template to the chromosome, achieving the desired DNA sequence modification. GT in plants has been challenging, and only a handful of cases have been reported in which endogenous plant genes have been successfully modified by HR [2–6]. One of the obstacles in achieving GT has been the ability to deliver sufficient donor templates to the plant cell to repair the DSB. Here, we demonstrate that this delivery barrier can be overcome using geminivirus-based DNA replicons (Fig. 1) to achieve high-frequency, targeted modification of the genome of an important crop plant, namely tomato.

## Results and discussion

**Fig. 2:**
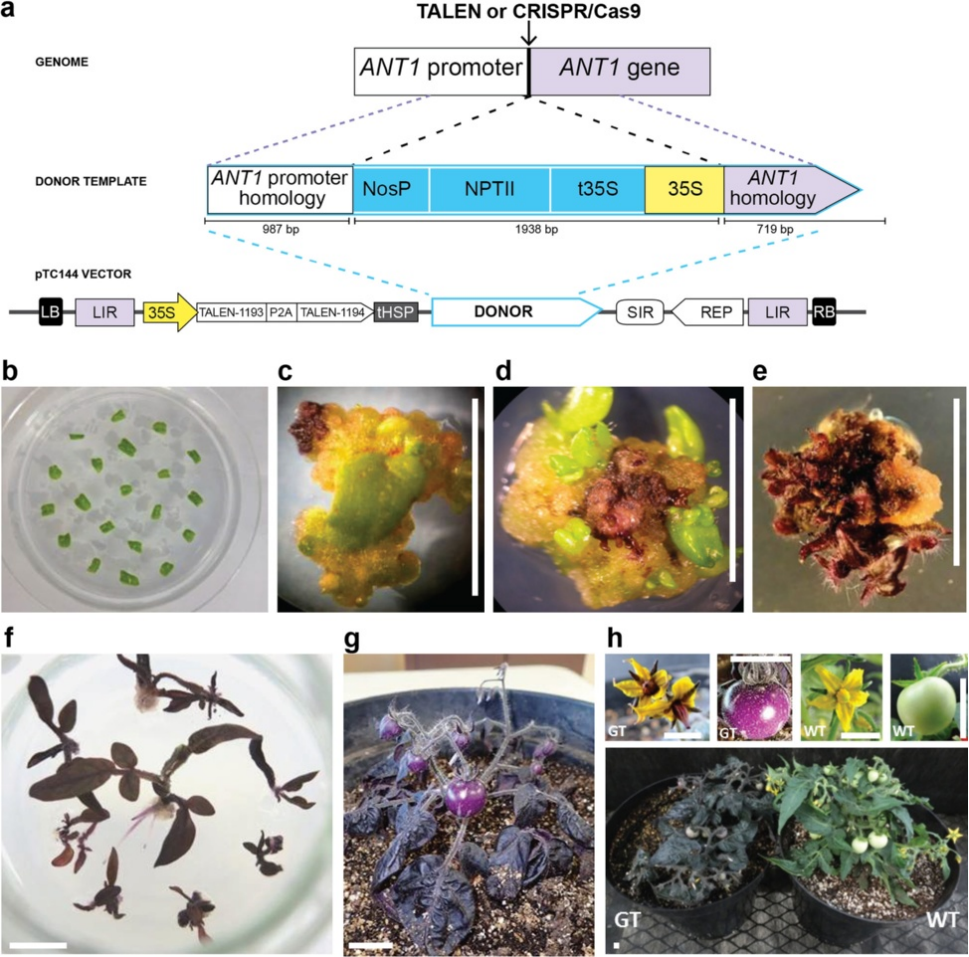
Gene targeting upstream of the *ANT1* gene. **a** *Top*: illustration of the GT event. Upon cleavage by the nuclease and homologous recombination with the replicon, the donor cassette is inserted upstream of *ANT1. Bottom*: structure of the transfer DNA (T-DNA) vector, pTC144, which produces DNA replicons. *LB* left T-DNA border, *LIR* BeYDV large intergenic region, *35S* cauliflower mosaic virus 35S promoter, *tHSP Arabidopsis thaliana* heat shock protein 18.2 terminator, *SIR* BeYDV short intergenic region, *REP* coding sequence for Rep/RepA, *RB* right T-DNA border. Additional components of the donor include: *NosP Agrobacterium tumefaciens* nopaline synthase promoter, *NPTII* neomycin phosphotransferase gene for kanamycin resistance, *t35S* CaMV 35S terminator. For expression of CRISPR/Cas9 reagents, the TALEN coding sequence was replaced with a plant codon-optimized Cas9 gene and the gRNAs were expressed from the AtU6 promoter (not shown). **b**–**h** Regeneration of tomato plants with targeted insertions. **b** Cotyledons of tomato cv. MicroTom after inoculation with *Agrobacterium*. **c** A recombinant explant 3 weeks after inoculation. Part of the developing callus accumulates anthocyanins due to the targeted promoter insertion and *ANT1* overexpression. **d** Explants 5 weeks after inoculation. Small shoots begin to develop on the purple callus. **e** Multiple shoots growing from the purple callus 10–12 weeks after inoculation. **f** Plantlets develop roots 12–14 weeks after inoculation. **g** Plantlet transplanted to soil. **h** Dark purple coloration in flowers, fruit and foliage results from targeted promoter insertion. Flowers, fruit and mature plants are compared between wild type (WT) plants and those that have undergone GT. Scale bars = 1 cm

Our target gene for modification in tomato was anthocyanin mutant 1 (*ANT1*). Overexpression of *ANT1*, which encodes a Myb transcription factor, results in intensely purple plant tissue due to anthocyanin accumulation [7, 8]. To achieve this phenotype through GT, we sought to insert the strong cauliflower mosaic virus 35S promoter upstream of the endogenous *ANT1* coding sequence (Fig. 2a). To this end, we designed two pairs of transcription activator-like effector nucleases (TALENs; 1193/1194 and 1195/1196) and two guide RNAs (gRNA7 and gRNA1b) to introduce DSBs at positions ranging from 56 bp to 203 bp from the *ANT1* start codon (Figure S1a, b in Additional file 1). Both TALENs showed activity in a single-strand annealing assay in tobacco protoplasts [9] (Figure S1c–e in Additional file 1). The 1193/1194 TALEN pair, which cut closest to the start codon, and the two gRNAs along with Cas9 were tested for their ability to create NHEJ-induced mutations at the target locus in tomato protoplasts (Figure S2 in Additional file 1). DNA encompassing the nuclease target sites was amplified by PCR and deep sequenced. The number of sequence reads with mutations ranged from 14 % for the TALEN pair to 29 % for gRNA1b.

To achieve GT, a single-component bean yellow dwarf virus (BeYDV) vector [10] was used to deliver both the donor template and cassettes encoding the nucleases (Fig. 2a). The TALENs or Cas9 were expressed from the 35S promoter, and the gRNAs were expressed from the AtU6 promoter. The donor template (Figure S3 in Additional file 1) consisted of the 35S promoter for targeted *ANT1* overexpression and a neomycine phosphotransferase II (NPTII) cassette to confer kanamycin resistance to recombinant cells (totaling 1938 bp). The 35S promoter and NPTII cassette were flanked by 987-bp and 719-bp homology arms. We expected that once delivered to the nucleus of a plant cell, the viral Rep protein would initiate circularization and rolling circle replication, resulting in hundreds to thousands of copies of the vector per cell. Indeed, when the BeYDV vector was delivered to tomato cells by *Agrobacterium*-mediated transformation, circularization of the geminivirus replicons was detected by PCR as early as 2 days post-inoculation and persisted for up to 8 weeks (Figure S4 in Additional file 1).

Cells that sustain a GT event should both accumulate anthocyanins from *ANT1* overexpression and be kanamycin resistant. In as little as 2 weeks after inoculation and growth on kanamycin-containing medium, we observed dark purple callus developing on several cotyledons (Fig. 2c). This visible phenotype allowed us to calculate the frequency of putative GT events, by dividing the number of cotyledons with one or more purple spots by the total number of inoculated cotyledons (Table 1, fourth column). The GT frequency was subsequently determined by normalizing the frequency of GT events by a baseline transformation frequency (Table 1, fifth column). The baseline transformation frequency was established by transforming cotyledons with a 35S::*ANT1* construct: 75 % of cotyledons (126 of 167) had one or multiple purple spots. To determine the GT frequency with BeYDV vectors encoding the TALEN pair 1193/1194, 1881 cotyledons were transformed (ten replicated experiments). A total of 137 purple calli were observed, resulting in a GT frequency of 9.65 × 10^−2^. Each of two experiments performed with clustered regularly interspaced short palindromic repeat (CRISPR)/Cas9 reagents produced comparable or lower GT frequencies (11.66 × 10^−2^ and 3.65 × 10^−2^).

**Table 1:** Gene targeting frequencies in tomato cotelydons

T-DNA construct	Number of transformed cotyledons	Purple calli	Number of purple calli/transformed cotelydons (%)	Gene targeting frequency
pTC147 (35S::ANT1 T-DNA)	167	126	75.45	NA
pTC144 (BeYDV with TALENs 1193/1194)	1881^a^	137	7.28	9.65 × 10^−2^
pTC217 (BeYDV with Cas9/gRNA1b)	216	19	8.80	11.66 × 10^−2^
pTC223 (BeYDV with Cas9/gRNA7)	218	6	2.75	3.65 × 10^−2^
pTC150 (BeYDV with donor-only)	200	0	0.00	<0.66 × 10^−2^
pTC206 (BeYDV with TALENs 1193/1194)^c^	183^b^	0	0.00	<0.72 × 10^−2^
pTC146 (BeYDV with donor, TALENs outside of the replicon)	199	7	3.52	4.67 × 10^−2^
pTC151 (Conventional T-DNA)	208	2	0.96	1.27 × 10^−2^
pTC208 (ToLCV with TALENs 1193/1194)	273^c^	8	2.93	3.88 × 10^−2^

^a^Total number of cotyledons transformed from ten independent experiments

^b^No selection was used in the callus induction medium

^c^Total number of cotyledons transformed from two independent experiments

*T-DNA* transfer DNA

Although the frequency of NHEJ-induced mutations was higher with CRISPR/Cas9 than with the TALEN (Figure S2 in Additional file 1), GT frequencies were comparable for both classes of reagents. A lack of correlation between frequencies of NHEJ-induced mutagenesis and GT was observed by others, for example, in human induced pluripotent stem cells [11, 12] and *Drosophila* [13]. The authors of these studies speculated the differences were due to the types of DSBs generated by TALENs (5′ overhangs) and CRISPR/Cas9 (blunt ends), which biased repair pathway choice [11, 12]. We believe that the differences in the frequencies and types of recombination events recovered are not related to our geminivirus-based method to induce gene targeting, but rather are due to intrinsic features of the NHEJ and HR repair pathways, which may be influenced by the types of DSBs made by TALENs and CRISPR/Cas9. Nevertheless, we demonstrate that both TALENs and CRISPR/Cas9 can be used to enhance GT in combination with geminivirus replicons.

In control experiments, no purple spots were observed among 200 cotyledons transformed with the donor-only (no nuclease) construct (GT frequency was <0.66 × 10^−2^), indicating that a DSB is essential to induce GT and that the truncated *ANT1* gene sequence in the right homology arm of the donor template does not produce functional ANT1 protein (Table 1). The GT frequency was not substantially altered if the nuclease was located on the transfer DNA (T-DNA) outside of the replicon and therefore unamplified. To determine if the GT frequency is enhanced using geminivirus replicons, we transformed cotyledons with a non-replicating T-DNA vector. Here, we observed a GT frequency of 1.3 × 10 ^−2^, which is approximately one order of magnitude less than the GT frequency observed with the BeYDV replicon.

In some instances, such as commercial crop production, the presence of the selectable marker in the genome is not desirable due to regulatory considerations, and so we also attempted to isolate GT events without selection. No purple spots were observed in the absence of kanamycin selection (Table 1), suggesting that the non-transformed green tissue outcompeted the purple cells, and that growth on kanamycin is required to give a selective advantage to cells that have undergone GT. The accumulation of anthocyanins may result in slower growth of the purple tissue compared with wild type (WT). Based on this observation, we anticipate that it might not be possible to regenerate plants with modifications that lead to growth inhibition without the use of a selectable marker, even when using alternative (potentially more efficient) transformation methods, such as biolistic bombardment, because the challenge lies in the regeneration of plants rather than transformation. *ANT1* was chosen as a target for modification because it allowed us to isolate and analyze the GT events at the callus stage, due to the purple pigmentation, and thus it served well for this proof-of-concept study in a crop species. Achieving gene targeting in plants without selection will require further optimization.

**Fig. 3:**
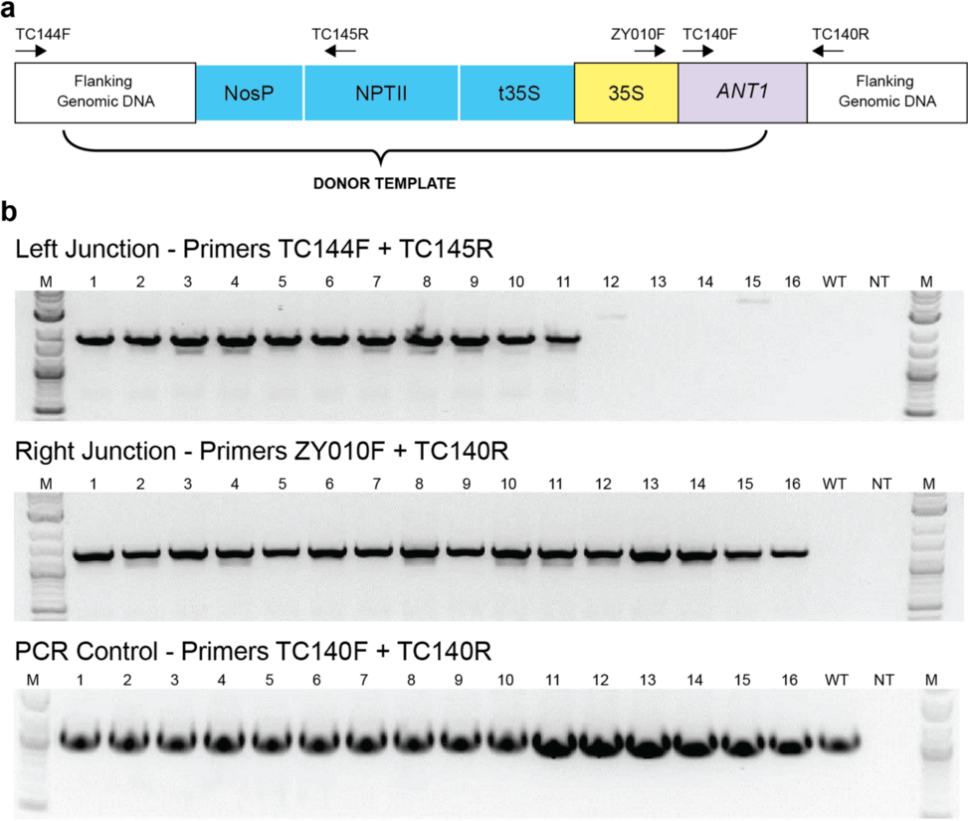
PCR analysis of targeted insertions in 16 purple calli obtained from one transformation experiment. **a** Diagram of the *ANT1* locus after gene targeting. *Numbered arrows* represent primers used in the study. **b** At the left junction, 11 of 16 purple calli gave the correct PCR product; 16 of 16 purple calli gave the correct product at the right junction. Products were obtained in all reactions with the PCR controls. Numbers represent purple calli corresponding to independent GT events. *M* 2-Log DNA ladder (New England Biolabs), *WT* wild type plant, *NT* no template control

To test whether the purple callus phenotype was due to precise GT of the *ANT1* locus, genomic DNA was isolated from 16 purple calli generated from an experiment using the BeYDV vector containing TALEN pair 1193/1194. PCR analysis demonstrated that all purple calli (16/16) had a right junction consistent with GT, and 11 of 16 gave PCR products of the predicted size for the left junction (Fig. 3). DNA sequencing revealed a perfect match to the expected sequence at the right and left junction in all but one sample, which had four nucleotide substitutions and one nucleotide insertion at the beginning of the *ANT1* gene (Figures S5 and S6 in Additional file 1). These results suggest that the purple calli indeed represent cells that have undergone GT, and furthermore, the purple calli consist of a high proportion of true and precise GT events (11 of 16 or 69 %).

**Fig. 4:**
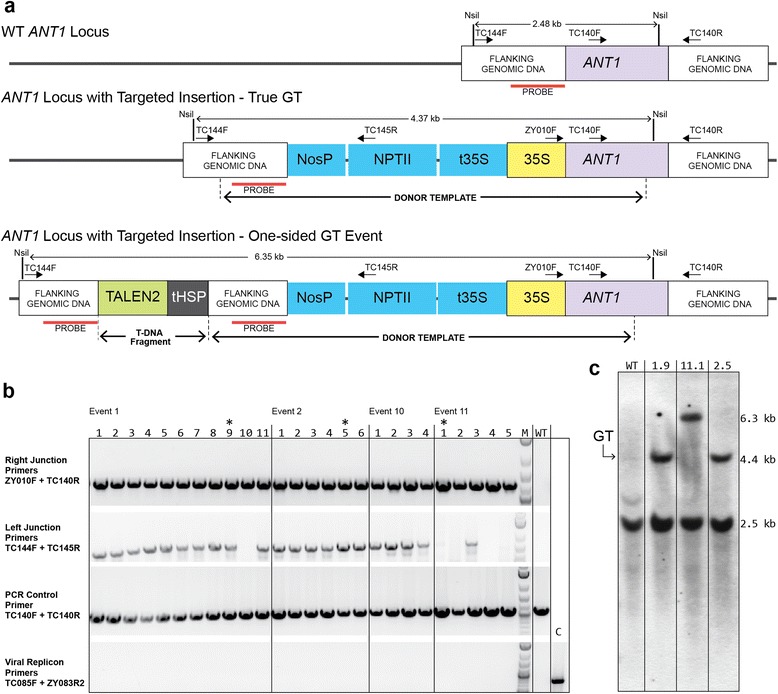
PCR and Southern blot analysis of GT events in pigmented plants. **a** Maps of the WT *ANT1* locus, the *ANT1* locus with a precise insertion, and an *ANT1* locus that has sustained a one-sided GT event. Primers used for PCR are indicated by *numbered arrows*. **b** PCR results from 26 purple plants recovered from four independently derived purple calli (events 1, 2, 10 and 11). PCR products of the expected size were obtained from all plants at the right junction. PCR products of the expected size of the left junction were obtained in all plants from events 2 and 10 and all plants from event 1 except for plant 1.10. Of the plants regenerated from event 11, only plant 11.3 proved positive for the left junction. Viral replicons were not detected in any of the mature plants. Primers used for detecting viral replicons were the same as in Fig. S4 in Additional file 1. *M* 2-Log DNA ladder (New England BioLabs), *WT* wild type plant, *C* positive control for virus circularization (genomic DNA from tissue 8 weeks after inoculation with the viral GT vector). Plants selected for Southern blot analysis are marked by *asterisks*. **c** Southern blot analysis of NsiI-digested genomic DNA from purple plants 1.9, 11.1 and 2.5. The 4.4-kb band in plants 1.9 and 2.5 is the size expected for precise insertion by HR. Plant 11.1 showed an approximately 6.3-kb band, indicative of a one-sided GT event. The 2.5-kb WT band was detected in all plants, demonstrating that they are heterozygous for the targeted insertion. No other bands were detected in any of the tested GT plants, suggesting that random integration of the T-DNA did not occur

Next, we sought to regenerate *ANT1*-modified plants from the purple calli. From three of the GT experiments using the BeYDV replicon, purple calli were regenerated into whole plants (Fig. 2b–h). This was accomplished by excising purple tissue 3–4 weeks after inoculation and then inducing regeneration without selection. A total of 72 whole plants were recovered from two calli from each of the first two experiments (events 1, 2, 10 and 11) and from one callus from the third experiment (event 14). Genomic DNA was prepared from all plants from the first two experiments, and PCR analysis was performed to assess the fidelity of recombination (Fig. 4). A pair of primers was designed to amplify the left and right recombinant junctions (Fig. 4a), and a band of the predicted size was recovered for the right recombinant junction in all the tested plants (Fig. 4b). Most plants also showed a band of the expected size for the left junction. DNA sequence analysis of the PCR products from plants 1.10, 2.5 and 11.1 revealed perfect repair by HR at the right junctions (Figure S7a in Additional file 1). The left junction of plant 1.10 was perfect; however, plant 2.5 had two single nucleotide substitutions, one at the left junction and one within the insertion cassette (Figure S7b in Additional file 1).

**Fig. 5:**
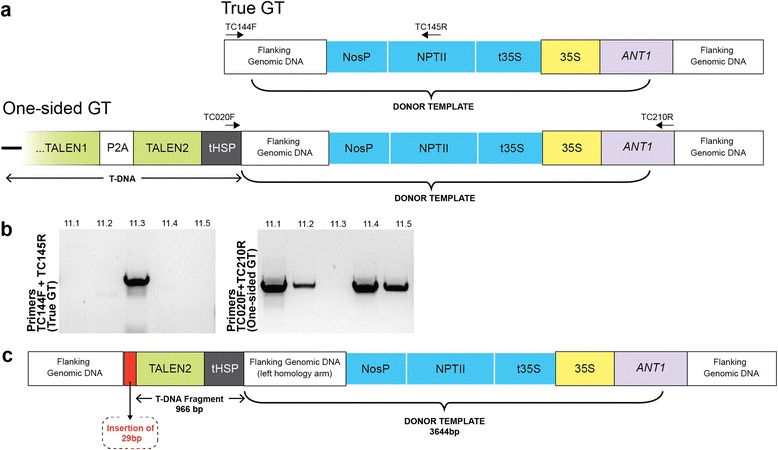
PCR detection of one-sided and true GT events in plants derived from event 11. **a** Diagrams of true and one-sided GT events. Primers used for PCR are marked with *numbered arrows*. **b** PCR analysis confirmed one-sided GT events in plants 11.1, 11.2, 11.4 and 11.5 and a true GT event in plant 11.3. **c** Reconstruction of the one-sided GT event from plant 11.1. DNA sequence analysis revealed precise, HR-mediated repair on the right side. On the left side, before re-ligation of the broken chromosome, an additional 966 bp of sequence was copied from the GT vector and another 29 bp of unknown origin

PCR analysis of the left junction failed to produce a product in the majority of plants derived from event 11 as well as from a plant derived from event 1 (Fig. 4b). We speculated that this might be the result of the non-conservative repair through synthesis-dependent strand annealing, in which HR is restricted to one side of the DSB and the other side is repaired by illegitimate recombination [14]. To test this hypothesis, a pair of primers was designed such that the forward primer annealed to the GT vector upstream of the left homology arm — DNA that would not be incorporated into the target locus if both ends of the DSB were repaired perfectly by HR. The reverse primer annealed to the genomic sequence just outside the right homology arm (Fig. 5a). These primers should only produce products from templates derived from such one-sided events. Indeed, we obtained specific products in all four plants from event 11 that initially failed to produce bands at the left junction, but not from plant 11.3, which gave a product for the left junction using the original set of primers (Fig. 5b). Sequencing of the PCR product from plant 11.1 revealed that, in addition to the donor cassette, 966 bp of sequence was copied from the GT vector and inserted at the *ANT1* locus. The junction with the tomato genomic DNA also had an additional 29 bp of sequence of unknown origin (Fig. 5c). DNA sequence of the right junction of the same plant confirmed precise repair by HR (Figure S7a in Additional file 1). Interestingly, even though all plants regenerated from each event were derived from the same piece of callus, events 1 and 10 produced plants that had undergone both one-sided and perfect HR. This could be explained if independent GT events occurred in two cells in close proximity, and the cells subsequently fused into a single mass of purple callus from which the plants were regenerated.

To further confirm the molecular nature of the GT events, we performed Southern blot analysis on plants 1.9, 11.1 and 2.5 (Fig. 4c), using a DNA probe that is homologous to sequences upstream of the *ANT1* start codon. In addition to detecting the GT event, this DNA probe was designed to also detect random T-DNA integration and extrachromosomal replicons. As expected, a 4.4-kb band indicative of true HR-mediated insertion was detected in plants 1.9 and 2.5, which gave the expected bands in the initial PCR survey. Plant 11.1, which was PCR-negative for the left junction, showed a larger ~6.3-kb band, consistent with a one-sided event. All plants showed a 2.5-kb band predicted for an unmodified locus, indicating the plants were heterozygous for the GT event. Remarkably we did not detect any additional bands in any of the four lines, suggesting that our modified plants were free of T-DNA insertions and extrachromosomal replicons. To confirm this observation, a second Southern blot was performed using different restriction enzymes (Figure S8 in Additional file 1). In this case, the T-DNA would be detected as a specific, 1.84-kb band, which was present in sample 11.1 but not in samples 1.9 and 2.5. Sample 11.1 carries a one-sided GT event that includes part of the T-DNA. Taking into account that plant 11.1 tested negative for T-DNA insertion in the first Southern blot and in PCR analysis (see below), we conclude that we did not find any evidence of random T-DNA integration in the whole plants.

Although in the majority of cases geminivirus replicons are released from the T-DNA by rolling-circle replication (not excision), in rare cases intramolecular recombination between the LIR repeats can lead to loss of the intervening sequence [15]. Random, off-target integration of such T-DNAs containing only an LIR would not be detected by the probe used in the above Southern blots. Therefore, both blots were re-probed with an LIR-specific probe (Figure S9 in Additional file 1). No signals were detected, suggesting that the genomes of these plants are free of such rare off-target integration events. To further confirm that no extrachromosomal replicons remained and no T-DNA insertions took place, we performed PCR using a pair of primers designed to amplify circular BeYDV genomes and another pair of primers designed to detect both the presence of replicons and random T-DNA insertions. No evidence of circular replicons was observed in any of the 26 mature plants recovered from events 1, 2, 10 and 11 using the first primer pair (Fig. 4b). Similarly, PCR performed with the other primer pair did not detect the presence of T-DNA or replicons in any of the five T0 plants tested (one from each GT event) or 34 T1 progeny (Figure S10 in Additional file 1). These results demonstrate that, unlike the *in planta* GT approach [16] in which the donor template is integrated into the genome, T-DNA integration is not required to achieve HR. Furthermore, our data indicate that the T-DNA simply serves as a vehicle for delivery and release of the viral replicons, and that T-DNA integration is dispensable. Although our *ANT1* overexpressing lines are transgenic due to the insertion of the 35S promoter, non-transgenic, replicon-free plants with precise DNA sequence modifications could be created by this approach.

Although we did not detect any off-target integration events, we were curious whether short indels were induced by NHEJ at other sites in the genome due to TALEN binding and cleavage. We used TAL Effector Nucleotide Targeter 2.0 [17] to identify the closest possible off-target sites for TALEN 1193/1194 in the tomato genome. As we used heterodimeric FokI architecture in our TALENs, which prevents cleavage of homodimeric targets [18], we focused on the three best off-target sites containing binding sites for each of the two different monomers. Two of these three sites had seven and six mismatches in the TALEN 1193 and 1194 binding sites, whereas the third had three and eight mismatches. All three sites were intergenic. We designed three pairs of primers (Table S1 in Additional file 2) and amplified these off-targets from genomes of five T0 plants (one plant from each GT event). The PCR products were subjected to a T7 endonuclease I (T7EI) assay and direct DNA sequencing. No mutations were found by either of these methods (Figure S11 in Additional file 1). The sensitivity of mutation detection by direct sequencing is 15–20 % [19], whereas the minimal detection limit for the T7EI assay was reported to be between 0.5 % and 5 % [20]. Thus, if any undetected mutations are present at these off-targets, their frequency should be below 5 %. With such a low frequency, the plants would have to be chimeras carrying both WT and mutant alleles, and the mutation would have to be induced at a later stage of development to be so rare, which is unlikely due to the fact that the nuclease-expressing replicons were detected in the transformed tissue up to 8 weeks post-inoculation, but not in mature plants (Fig. 4b; Figure S4b in Additional file 1). Furthermore, it is very unlikely that such rare mutations would be transmitted to progeny [21]. We conclude that our approach resulted in clean GT lines with no detectable off-target mutations.

**Fig. 6:**
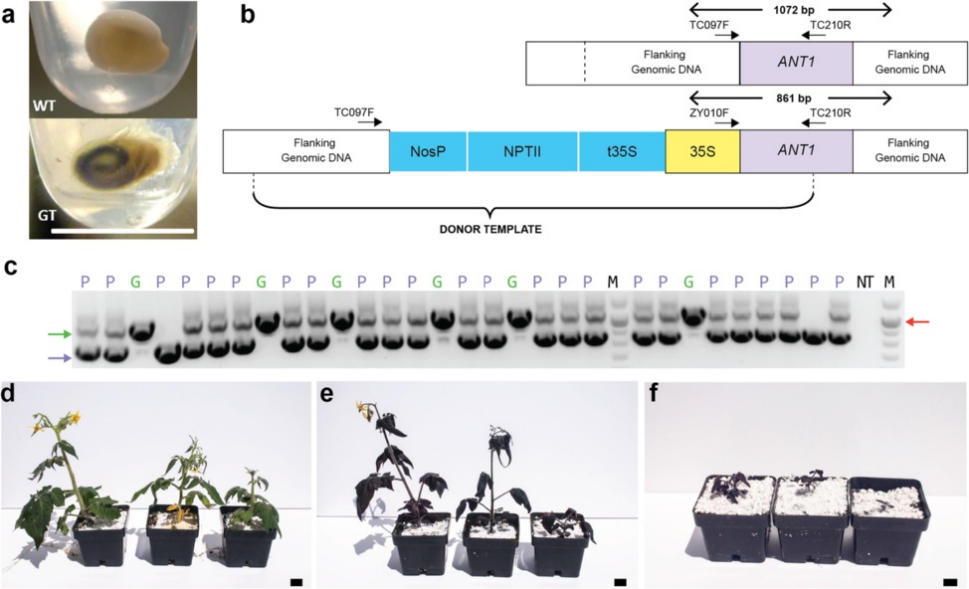
Transmission of the targeted insertion to the next generation. **a** Purple coloration is visible in the embryos within the seeds. **b** Scheme of the multiplexed PCR used to detect both WT and GT events in progeny of GT lines. Primers TC097F, ZY010F and TC210R (marked by *arrows*) were used in a single reaction. **c** A sample gel picture with products from PCR analysis of 30 T1 seedlings (gel pictures from PCR analysis of all 175 screened seedlings are provided in Fig. S12 in Additional file 1). All three possible genotypes were detected. *Green arrow* marks the WT products, the *purple arrow* the GT products, and *red arrow* the 1.0-kb band in the DNA ladder. The phenotype of each seedling is marked by *P* (purple) or *G* (green). *M* 2-Log DNA ladder (New England Biolabs), *NT* no template control. **d–f** Pictures of three of each homozygous WT (**d**) and heterozygous (**e**) and homozygous (**f**) GT T1 plants. The homozygous GT plants have reduced growth due to excessive accumulation of anthocyanins. Scale bars = 1 cm

To test whether the targeted DNA insertions were heritable, we analyzed progeny of 24 plants regenerated from events 1, 2, 11 and 14 (Fig. 6 and Table 2). A total of 123 T1 seedlings showed the characteristic purple color, which was already visible at the embryo stage within the seed (Fig. 6a). PCR analysis confirmed that 100 of these seedlings (57.1 %) were heterozygous and 23 (13.1 %) were homozygous for the promoter insertion; the other 52 green seedlings were WT (Table 2; Figure S12 in Additional file 1). Collectively, 70.2 % of the progeny were purple and 29.7 % were green. These data are consistent with the T0 plants being heterozygous for the targeted modification: all but 5 of the 24 plants segregated green progeny, and of these five, only a few seeds were produced. The number of plants carrying the modified *ANT1* allele in the homozygous state was slightly lower than the expected 1:2:1 segregation frequency. This could be caused by a growth inhibitory effect resulting from excessive accumulation of anthocyanins [8]. Growth inhibition was observed to be much stronger in the homozygous plants (Fig. 6f) than the heterozygotes (Fig. 6e), the latter of which grew comparably to WT (Fig. 6d). It is possible that seed viability/germination is also affected by the excess pigments, which would result in the observed underrepresentation of homozygous *ANT1* overexpressing plants in the T1 progeny. Furthermore, this inhibitory effect might also have been the reason why homozygous plants were not recovered in the T0 generation. We indeed observed that many potentially homozygous purple calli did not regenerate shoots; however, due to the small size of the calli, we could not test whether they were homozygous or not, as we could never be 100 % sure that only purple tissue was excised without a few WT cells from the surrounding, non-transformed tissue, which would subsequently cause all the samples to look like heterozygotes when analyzed by PCR. Therefore, to test this hypothesis, we conducted an experiment in which we directly tested the regenerative capacity of homo- and heterozygous tissue derived from the cotyledons of PCR-genotyped T1 seedlings. We did not find any difference between the samples in terms of callus and shoot induction (Figure S13 in Additional file 1). Thus, it remains unclear why homozygous plants were not regenerated in the T0 generation, and it may simply be that the frequency of HR is too low to recover bi-allelic events in the small number of plants generated. Importantly, we did demonstrate that plants homozygous for the insertion can be recovered in the T1 generation, and these results collectively demonstrate that our approach generates heritable genomic modifications.

**Table 2:** Segregation of the purple phenotype in T1 progeny

Event	Plant	Green progeny	Purple progeny	Purple progeny	Total
		WT/WT	P/WT	P/P	
1	1.4	4	2	0	6
	1.5	8	20	0	28
	1.6	2	0	0	2
	1.7	3	5	0	8
	1.9	1	1	0	2
		18 (39.1 %)	28 (60.9 %)	0 (0.0 %)	46
2	2.6	1 (33.3 %)	2 (66.6 %)	0 (0.0 %)	3
11	11.3	2 (50 %)	1 (25 %)	1 (25 %)	4
14	14.1	1	3	3	7
	14.2	1	3	0	4
	14.3	3	7	2	12
	14.4	7	8	4	19
	14.5	3	7	0	10
	14.6	4	7	5	16
	14.7	1	4	4	8
	14.8	1	5	1	7
	14.9	5	10	2	17
	14.10	3	6	1	10
	14.11	0	2	0	2
	14.15	0	2	0	2
	14.28	1	1	0	2
	14.35	0	1	0	1
	14.38	1	1	0	2
	14.40	0	1	0	1
	14.48	0	1	0	1
		31 (25.4 %)	69 (56.6 %)	22 (18.0 %)	122
Total		52 (29.7 %)	100 (57.1 %)	23 (13.1 %)	175

We hypothesized that GT frequencies may be further increased using a geminivirus for which tomato is the primary host. To this end, we constructed another GT vector based on the DNA-A component of tomato leaf curl virus (ToLCV; *Begomovirus* [22]; Figure S14 in Additional file 1). We engineered the ToLCV vectors to contain the same TALEN pair and donor molecule as used in the BeYDV vectors. Whereas we observed purple calli with the ToLCV vector, the GT frequency was lower than with the BeYDV vector (Table 1). This could be explained by differences in rates of replication of the two viruses [23] or other factors such as the expression of three *Begomovirus-*specific proteins, AC2 (TrAP), AC3 (Ren) and AC4, which facilitate ToLCV replication.

## Conclusions

Precise gene editing in crop species provides an alternative to traditional transgenesis, in which foreign DNA is inserted into a plant genome to create a trait of value [24, 25]. Whereas both gene editing and transgenesis accelerate trait development, gene editing, in some instances, leads to crops with no foreign DNA, and thus may pose fewer regulatory hurdles for cultivar deployment. A few crop varieties have already been produced using site-specific nucleases to create targeted mutations through imprecise repair of breaks by NHEJ [26–29]. To fully exploit the potential of gene editing, however, efficient methods for the precise modification of genes will be needed — for example, to create new alleles by introducing point mutations in cases where a gene knock-out is not desirable. Our study provides an example of such a method to create tomato plants modified by GT. Compared with other GT approaches in plants [6, 16], the use of geminivirus replicons creates genome-modified plants without the need for stable integration of transgenes, which would have to be segregated away in subsequent generations to produce non-transgenic plant lines. We show that geminivirus vectors are efficient tools for GT in tomato, and coupled with TALENs or CRISPR/Cas9 reagents, they allow the targeting of virtually any sequence in a given genome, making it possible to extend this technology to other crop species to create valuable traits.

## Materials and methods

### Vector construction

All BeYDV-based geminivirus vectors used in this study were derived from pLSLR [10], a T-DNA vector (pCAMBIA1300) that contains the BeYDV (accession DQ458791 [30]) Rep/RepA, long intergenic region (LIR) and short intergenic region (SIR) in an LIR-SIR-Rep/RepA-LIR orientation. pLSLR was modified to create a universal BeYDV GT vector for cloning of custom donor templates and TALENs created with our Golden Gate TALEN assembly kit [31] or CRISPR/Cas9 reagents. The TALEN expression cassette consists of a 35S promoter and two N152/C63 truncated TALEN backbones. The TAL effector repeats were replaced by a *ccdb* gene (flanked by Esp3I sites in the first TALEN) and a *lacZ* gene (flanked by BsaI sites in the second TALEN). The TALEN coding sequences were separated by the P2A ribosomal skipping sequence and followed by the heat shock protein 18.2 transcriptional terminator. The Cas9 expression cassette consists of a 35S promoter and a plant codon-optimized Cas9 coding sequence described in Fauser et al*.* 2014 [32]. The TALEN or Cas9 expression cassettes were inserted between the upstream LIR and SIR sequence or outside the replicon borders of pLSLR by Gibson assembly [33]. The resulting vectors were named pTC110 (TALEN cassette within the replicon) and pTC111 (TALEN cassette outside the replicon). The BeYDV CRISPR/Cas9 GT vectors, pTC217 and pTC223, express gRNA1b and gRNA7, respectively.

To create our *ANT1* GT vector, the hygromycin resistance cassette was first removed from the pTC110 and pTC111 backbones, and the TALENs were cloned into the Esp3I and BsaI cloning sites, giving rise to pTC130 and pTC131. The donor template was cloned by Gibson assembly of PCR fragments containing the left *ANT1* homology arm, the nopaline synthase (NOS) promoter, the NPTII gene for kanamycin resistance, a 35S polyA sequence, the 35S promoter, and the right *ANT1* homology arm. The donor template was inserted into the BaeI site between the heat shock protein (HSP) terminator and the SIR in pTC130 and pTC131. The GT vector without kanamycin selection (pTC206) was constructed accordingly, but fragments containing the NOS promoter, the NPTII gene and the 35S polyA sequence were omitted. The final *ANT1* GT vectors were named pTC144 (TALEN cassette in the replicon) and pTC146 (TALEN cassette outside the replicon). The CRISPR/Cas9 GT vectors pTC217 and pTC223 have the same donor template; however, they carry the Cas9 coding sequence and gRNA1b and gRNA7, respectively. All primers used for vector construction are listed in Table S1 in Additional file 2.

The control vector without the nuclease, pTC150, was created by removing the TALEN cassette by AscI/PmlI digestion and re-ligation after creating blunt ends. The control non-viral GT vector, pTC151, was created by removing the SIR, Rep/RepA and downstream LIR from pTC144 by SwaI/PmeI digestion and re-ligation. To create the 35S:*ANT1* transformation control vector, pTC147, the *ANT1* gene was amplified using primers TC127F and TC079R (Table S1 in Additional file 2) and tomato cv. MicroTom genomic DNA as a template, and then Gibson-assembled into NcoI/BstEII-digested pCAMBIA1302. The hygromycin resistance cassette was removed by BstXI/PspXI cleavage and replaced with the BstXI/PspXI fragment containing the kanamycin resistance cassette from pCAMBIA2300.

The ToLCV GT vector (pTC208) is similar in structure to the BeYDV vector, except the BeYDV LIRs, SIR and Rep/RepA were replaced with ToLCV CR on one side and the CR-AC3 region on the other side of the replicon, as described in Pandey et al*.* [22].

Vector maps and sequences can be found in Additional files 3, 4, 5, 6, 7, 8, 9, 10 and 11. The list of all vectors used in this study is in Table S2 in Additional file 2. All vectors will be made publicly available at Addgene [34] (plasmid #70006, 70012–70019).

### TALEN activity in protoplasts

Protoplast isolation, transformation and flow cytometry analyses were done as described in Zhang et al*.* [9]. TALENs were cloned into a pCLEAN-G vector [35] in the p35S:TALEN1:P2A:TALEN2:tHSP configuration. The respective TALEN targets were cloned into pZHY705, a derivative of pZHY402 [9] that has a 120-bp internal yellow fluorescent protein (YFP) sequence duplication. The two plasmids were co-transformed into tobacco protoplasts for flow cytometry analyses. Only the TALEN expression vector or Cas9 and gRNA expressing vectors were co-transformed into tomato protoplasts for screening of TALEN/CRIPSR-Cas9-induced mutations at the *ANT1* target locus. Genomic DNA isolated from ~200,000 cells 2 days after transformation was used as a template for the deep sequencing library preparation.

### Amplicon library preparation and deep sequencing

Amplicon libraries were prepared by two-step PCR according to the Illumina protocol for 16S metagenomic sequencing library preparation. In the first step, a 340-bp region of the *ANT1* locus, including the TALEN1193/1194, gRNA1b and gRNA7 target sites, was PCR-amplified with primers TC097_ampli_F2 and TC097_ampli_R (Table S1 in Additional file 2), which have overhangs complementary to Nextera XT indices. Protoplast genomic DNA (25 ng) was used as template. PCR products were purified with 1.8× volume of Agencourt AMPure XP Beads (Beckman Coulter, Brea, USA) and eluted into 50 μl of 10 mM Tris pH 8.5. The purified PCR product (5 μl) was used as template for the second PCR to attach dual indices and Illumina sequencing adapters. PCR products were purified using 50 μl of Agencourt AMPure XP Beads (Beckman Coulter) and eluted into 25 μl of 10 mM Tris pH 8.5. Purified and quantified amplicons were mixed in equimolar amounts. The final pooled library was sequenced on Illumina MiSeq flowcell with MiSeq reagent Nano kit v2 (Illumina). Paired-end sequencing was performed using 251 cycles.

### Sequencing data analysis

The quality of sequencing reads was verified in FastQC [36] . Read trimming was done with Trimmomatic-0.32 [37] using the following parameters: ILLUMINACLIP:nextera_xt_indexis.fa:2:30:10 LEADING:30 TRAILING:30 SLIDINGWINDOW:4:20 HEADCROP:0 MINLEN:80. Next, forward and reverse trimmed reads were merged by SeqPrep [38] with default parameters. Merged reads for each sample were mapped to the reference sequence using Geneious R7 mapper [39] in custom sensitivity mode (allow gaps, 80 %; maximum gap size, 500 bp; maximum mismatches per read, 5 %). Mapped reads were trimmed along the nuclease target site and exported in bam format. Bed files with CIGAR string were generated from bam files using Bedtools v.2.17.0 [40]. A custom bash script was used to select all unique indel variants and their counts. All unique reads with deletions were mapped again onto the reference sequence in Geneious and manually verified to make sure they span the nuclease target site. Unique reads containing insertions were aligned to the reference sequence by Mafft aligner implemented in Geneious R7 [39]. Finally, these verified reads were used to calculate the frequencies of NHEJ-induced mutagenesis for individual nucleases.

### *Agrobacterium* preparation

*Agrobacterium tumefaciens* strain LBA4404 containing each binary vector was grown in YENB medium (7.5 g Bacto yeast extract, 5 g Bacto beef extract and 3 g Bacto peptone in 1 L distilled water) supplemented with 50 mg/L kanamycin. Two days before transformation, a single colony was used to initiate a 2-ml culture and incubated at 28 °C in a shaking incubator. The following day, 50–2000 μl of the initial culture was used to start a 50-ml culture and incubated overnight at 28 °C. On the day of transformation, the OD_600_ was adjusted to 0.8 as in Van eck et al*.* [41]. The culture was spun down and resuspended in 50 ml of MS liquid medium [41] with addition of 100 μM acetosyringone.

### Plant transformation and regeneration

*A. tumefaciens*-mediated transformation of tomato cultivar MicroTom was performed according to Van Eck et al*.* [41] with some modifications. Seeds were surface sterilized by shaking in 50 % bleach for 10 min followed by three rinses with sterile water. They were then germinated on ½ MSO media at 25 °C in the dark for 3 days and grown for 6 days under a 16-h photoperiod. Cotyledons were isolated from these 9-day-old seedlings, and the distal and proximal tips were removed. The cotyledons were then gently poked using a sterile insulin syringe needle and placed on plates with modified KCMS media with 0.5 mg/L indolyl acetic acid (IAA) instead of 2,4D and 100 μM acetosyringone. No feeder layer was used. *Agrobacterium* inoculation was done on the day of cotyledon isolation. After 48-h co-cultivation in the dark, explants were placed on non-selective (without kanamycin) 2Z plates (all zeatin-containing medium was prepared with 400 mg/L timentin and 0.1 mg/L IAA) and cultivated under a 16-h photoperiod. Five days later, explants were transferred to selective 2Z plates with 100 mg/L kanamycin and cultivated for 2 weeks or until purple tissue appeared. The purple tissue was separated from the explants, placed on 1Z non-selective plates, and 2 weeks later on 0.5Z non-selective plates. Then, the explants were transferred to fresh non-selective shooting media (same as 2Z, but zeatin was replaced with 0.1 mg/L gibberellic acid) every 2 weeks until shoots appeared. Shoots were excised from the callus, transferred to non-selective rooting medium and cultivated until they developed roots. Finally, rooted plantlets were transferred to soil-less potting mix and cultivated in a growth chamber or in a greenhouse as described [41].

### PCR genotyping

Genomic DNA was extracted from purple callus tissue or leaves of young plantlets using the DNeasy Plant Mini Kit (QIAGEN). Using the primers listed in Table S1 in Additional file 2, samples were genotyped for the presence of the right and left recombination junctions, as well as one-sided recombinant products and virus circularization. All PCR products were resolved on 1 % agarose gels. Selected PCR products were excised, purified, cloned into the pJET1.2 vector (Thermo Fischer Scientific) and sequenced. Sequences were analyzed using Geneious R7 [39]. PCR genotyping of T1 progeny was done by multiplex direct PCR with the Phire Plant Direct PCR Master Mix (Thermo Scientific) using the Dilution & Storage protocol. To detect both WT and insertion alleles, primer TC210R (Table S1 in Additional file 2), which anneals to the *ANT1* gene outside of the donor homology was combined with TC097F, which anneals to the native sequence upstream of *ANT1* start codon, and primer ZY010F, which anneals to the 35S promoter.

### Southern and dot blot analysis

Genomic DNA was extracted from 1 g of young leaf tissue as described by Ince et al*.* [42]. NsiI- or BspHI and EcoRV-digested genomic DNA (50 μg) was resolved on a 0.8 % agarose gel and blotted by capillary transfer onto Hybond N+ membrane (GE Healthcare). For dot blots, 100 ng of plasmid DNA was pipetted and UV crosslinked to the membrane. A PCR product amplified with *ANT1*-specific primers TC080F and C2R or LIR primers TC101F and TC246R (Table S1 in Additional file 2) was used as a probe. Purified PCR product (200 ng) was labeled using the Amersham AlkPhos Direct Labeling and Detection System (GE Healthcare) and hybridized to membranes at 60 °C overnight. Membranes were processed according to the manufacturer’s recommendations. Probes were detected using the Amersham CDP-Star Detection Reagent (GE Healthcare), and signals were captured on X-ray film (Amersham Hyperfilm ECL, GE Healthcare). For re-probing, membranes were stripped in 0.5 % SDS solution at 60 °C.

### Off-target analysis

TALEN1193/1194 off-target sites were identified using the Paired Target Finder function of TAL Effector Nucleotide Targeter 2.0 [17]. The default search criteria did not return any heterodimeric off-target sites. Changing the score cutoff value to 4.0 yielded a list of 220 sites with low scores. Three sites out of this list were chosen with best scores and most optimal spacer lengths. Off-target sites were PCR amplified with the primers in Table S1 in Additional file 2. The PCR products were purified using the QIAquick PCR purification kit (QIAGEN) and directly sequenced or subjected to T7 endonuclease analysis. The T7 assay was performed according to the manufacturer’s protocol. Briefly, 200 ng of each PCR product was mixed with 1× NEBuffer 2 (NEB), denatured 5 min at 95 °C and gradually cooled down in a PCR machine. T7 endonuclease (1 μl) was added to the samples followed by incubation 15 min at 37 °C and electrophoresis on 1 % agarose gels.

### Availability of supporting data

The deep sequencing data is available under the European Nucleotide Archive (ENA) accession [ENA:PRJEB10891] [43].

## Additional files

Additional file 1: Figure S1. Design of nucleases and functional testing in tobacco protoplasts. **Figure S2.** Mutagenesis of the *ANT1* locus as measured by deep sequencing. **Figure S3.** Sequence of the donor template for targeted insertion into the *ANT1* locus. **Figure S4.** PCR-based detection of circularized viral replicons in *Agrobacterium*-inoculated tomato explants. **Figure S5.** DNA sequences at the left recombination junction in 11 of 16 purple calli obtained from one transformation experiment. **Figure S6.** DNA sequences at the right recombination junction in 16 purple calli obtained from one transformation experiment. **Figure S7.** DNA sequence analysis of the recombination junctions in three selected purple plants, one each from events 1, 2 and 11. **Figure S8.** Southern blot analysis of BspHI + EcoRV digested genomic DNA from three selected purple plants (same as in Fig. 4). **Figure S9.** Detection of viral LIR sequences by Southern blot analysis of NsiI and BspHI + EcoRV digested genomic DNA from three selected purple plants. **Figure S10.** PCR analysis of random T-DNA integration. **Figure S11.** Analysis of three closest off-targets for TALEN1193/1194 in five independent GT events. **Figure S12.** Multiplexed PCR analysis of 175 T1 progeny from four independent GT events (1, 2, 11 and 14). **Figure S13.** Regenerative capacity of plants homo- and heterozygous for targeted promoter insertions. **Figure S14.** Structure of the ToLCV gene targeting T-DNA vector. (PDF 2387 kb) Additional file 2: Table S1. List of primers used in the study. **Table S2.** List of constructs used in the study. (PDF 45 kb) Additional file 3:
**Annotated sequence of the vector pTC144.** (GB 53 kb) Additional file 4:
**Annotated sequence of the vector pTC146.** (GB 49 kb) Additional file 5:
**Annotated sequence of the vector pTC147.** (GB 20 kb) Additional file 6:
**Annotated sequence of the vector pTC150.** (GB 28 kb) Additional file 7:
**Annotated sequence of the vector pTC151.** (GB 43 kb) Additional file 8:
**Annotated sequence of the vector pTC206.** (GB 43 kb) Additional file 9:
**Annotated sequence of the vector pTC208.** (GB 43 kb) Additional file 10:
**Annotated sequence of the vector pTC217.** (GB 33 kb) Additional file 11:
**Annotated sequence of the vector pTC223.** (GB 33 kb)

## Abbreviations

ANT1: anthocyanin mutant 1
BeYDV: bean yellow dwarf virus
CRISPR: clustered regularly interspaced short palindromic repeat
DSB: double-strand break
gRNA: guide RNA
GT: gene targeting
HR: homologous recombination
HSP: heat shock protein
IAA: indolyl acetic acid
LIR: long intergenic region
NHEJ: non-homologous end joining
NOS: nopaline synthase
NPTII: neomycine phosphotransferase II
PCR: polymerase chain reaction
SIR: short intergenic region
TALEN: transcription activator-like effector nuclease
T-DNA: transfer DNA
ToLCV: tomato leaf curl virus
WT: wild type
