# Supplementary material for: High-frequency, precise modification of the tomato genome
Source: Genome Biol. 2015 Nov 6;16:232. doi: 10.1186/s13059-015-0796-9 (PMC4635538; doi:10.1186/s13059-015-0796-9)
Supplement: Additional file 1: Figure S1. — Design of nucleases and functional testing in tobacco protoplasts. Figure S2. Mutagenesis of the ANT1 locus as measured by deep sequencing. Figure S3. Sequence of the donor template for targeted insertion into the ANT1 locus. Figure S4. PCR-based detection of circularized viral replicons in Agrobacterium-inoculated tomato explants. Figure S5. DNA sequences at the left recombination junction in 11 of 16 purple calli obtained from one transformation experiment. Figure S6. DNA sequences at the right recombination junction in 16 purple calli obtained from one transformation experiment. Figure S7. DNA sequence analysis of the recombination junctions in three selected purple plants, one each from events 1, 2 and 11. Figure S8. Southern blot analysis of BspHI + EcoRV digested genomic DNA from three selected purple plants (same as in Fig. 4). Figure S9. Detection of viral LIR sequences by Southern blot analysis of NsiI and BspHI + EcoRV digested genomic DNA from three selected purple plants. Figure S10. PCR analysis of random T-DNA integration. Figure S11. Analysis of three closest off-targets for TALEN1193/1194 in five independent GT events. Figure S12. Multiplexed PCR analysis of 175 T1 progeny from four independent GT events (1, 2, 11 and 14). Figure S13. Regenerative capacity of plants homo- and heterozygous for targeted promoter insertions. Figure S14. Structure of the ToLCV gene targeting T-DNA vector. (PDF 2387 kb) [file 13059_2015_796_MOESM1_ESM.pdf]

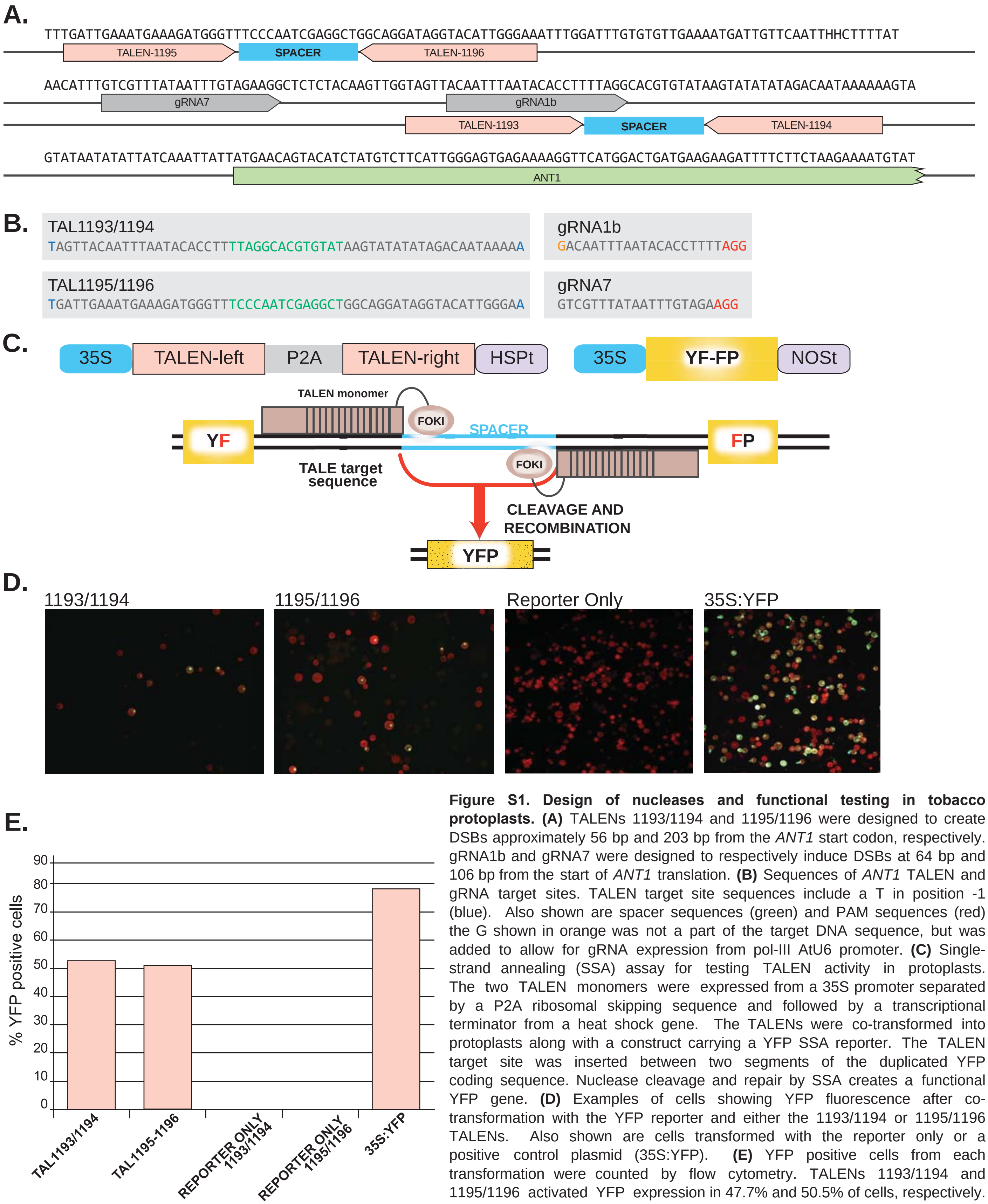

Figure S1. Design of nucleases and functional testing in tobacco protoplasts. (A) TALENs 1193/1194 and 1195/1196 were designed to create DSBs approximately 56 bp and 203 bp from the *ANT1* start codon, respectively. gRNA1b and gRNA7 were designed to respectively induce DSBs at 64 bp and 106 bp from the start of *ANT1* translation. **(B)** Sequences of *ANT1* TALEN and gRNA target sites. TALEN target site sequences include a T in position -1 (blue). Also shown are spacer sequences (green) and PAM sequences (red) the G shown in orange was not a part of the target DNA sequence, but was added to allow for gRNA expression from pol-III AtU6 promoter. **(C)** Single-strand annealing (SSA) assay for testing TALEN activity in protoplasts. The two TALEN monomers were expressed from a 35S promoter separated by a P2A ribosomal skipping sequence and followed by a transcriptional terminator from a heat shock gene. The TALENs were co-transformed into protoplasts along with a construct carrying a YFP SSA reporter. The TALEN target site was inserted between two segments of the duplicated YFP coding sequence. Nuclease cleavage and repair by SSA creates a functional YFP gene. **(D)** Examples of cells showing YFP fluorescence after co-transformation with the YFP reporter and either the 1193/1194 or 1195/1196 TALENs. Also shown are cells transformed with the reporter only or a positive control plasmid (35S:YFP). **(E)** YFP positive cells from each transformation were counted by flow cytometry. TALENs 1193/1194 and 1195/1196 activated YFP expression in 47.7% and 50.5% of cells, respectively. This compares to 78.1% for the 35S:YFP control. No YFP-positive cells were detected in the controls where only the reporter construct with either 1193/1194 or 1195/1196 TALEN target were used for transformation.

	TALEN 1193/1194		gRNA1b		gRNA7		WT	
**Input Reads**	31782		23797		21071		26994	
**Deletions**	2762		4278		1562		9	
**Insertions**	1772		2678		3746		2	
**Total Indels**	4534	14.27 %	6956	29.23 %	5318	25.24 %	11	0.04 %

Figure S2. Mutagenesis of the *ANT1* locus as measured by deep sequencing. Tomato protoplasts were transformed with either a vector containing 1) the two TALEN monomers expressed from a 35S promoter and separated by a P2A ribosomal skipping sequence or 2) two seperate vectors - a 35S:Cas9 construct and an AtU6:gRNA construct. Genomic DNA was extracted 48h later and the target site amplified by PCR. The PCR amplicon was subjected to deep sequencing using the Illumina platform. Mutation frequency was determined as the number of reads containing indels per number of input reads. WT, water was used instead of plasmid DNA for protoplast transformation.

ACCACAACACTTGTCGGTGAGATTATTTAATGCTGATTAGATTAGACAAAAATTAATTAGTTTTGAGTAGTGGCGTAAGTGTAAATAATTAGTCTCTTTTTTAACTT
AGAAAATAGTTTAATCCTTAGTATAAATAGTCAAAATCACTGGAATGAAAAACAGTTTTTAATTTTTCCAAATTTGATTCTGATACCATGTTAAATTCGTGGTTCAA
AATCACTGCAATGAAAAGAGCAATATTGTTTAACTTTTTTTAGGAAAATCGAATTGATTTATAGTCAGTTGATATAGAGTGAATACATAAGGAACATATACAGTTGA
TACAATTGTATAATTCGTTCATACACTTAATACAAAGTGAACCCACAAGGAACATATACACTTAATATAATTGTATTCCTTGATACAAACCAATTTTGTTCGTGTCT
CTACTCTCTATTTCAATTTCGCTTGACTCTTTACTTTTTCTAATATGTAGCTATAAATCGTAATTAAACAATACTATATCTCTAAATCTCTTATTAAGCTCAAACTA
TGGTCATATTCGAAAAAATCCTTTTAAATATTGGTCCCCTCTCACGATTAATGATAGTTATAACTAACATTCAAATTTTAGTTGTACTTGACATCTAAAACTTAAAA
AATAGTACAAGTTAACTTTTTCTTTTTTTTAAAAAAAGGAAATACTTGTATTTATTTTTTTAATATATAGTTATATTTTTGGTTATTTGAAAATACTTGATCTGTCA
TGTATGCTCAGTTAAATATCGTCACATTATAGAGAAAAAAGTAATAGGAGAAAAAAATTAAAAATTATTTCGAAAAATCAAAATTTTTTTTTGATTGAAATGAAAGA
TGGGTTTCCCAATCGAGGCTGGCAGGATAGGTACATTGGGAAATTTGGATTTGTGTGTTGAAAATGATTGTTCAATTTGGCTTTTATAACATTTGTCGTTTATAATT
TGTAGAAGGCTCTCTACAAGTTGGACTGAAGGCGGGAAACGACAATCTGATCATGAGCGGAGAATTAAGGGAGTCACGTTATGACCCCCGCCGATGACGCGGGACAA
GCCGTTTTACGTTTGGAACTGACAGAACCGCAACGTTGAAGGAGCCACTCAGCCGCGGGTTTCTGGAGTTTAATGAGCTAAGCACATACGTCAGAAACCATTATTGC
GCGTTCAAAAGTCGCCTAAGGTCACTATCAGCTAGCAAATATTTCTTGTCAAAAATGCTCCACTGACGTTCCATAAATTCCCCTCGGTATCCAATTAGAGTCTCATA
TTCACTCTCAATCCAAATAATCTGCACCGGATCCGCTAGAGGATCTCGACCTGCAAGATCCCGGGGGGCAATGAGATCCTAGGATGGGGATTGAACAAGATGGATTG
CACGCAGGTTCTCCGGCCGCTTGGGTGGAGAGGCTATTCGGCTATGACTGGGCACAACAGACAATCGGCTGCTCTGATGCCGCCGTGTTCCGGCTGTCAGCGCAGGG
GCGCCCGGTTCTTTTTGTCAAGACCGACCTGTCCGGTGCCCTGAATGAACTCCAGGACGAGGCAGCGCGGCTATCGTGGCTGGCCACGACGGGCGTTCCTTGCGCAG
CTGTGCTCGACGTTGTCACTGAAGCGGGAAGGGACTGGCTGCTATTGGGCGAAGTGCCGGGGCAGGATCTCCTGTCATCTCACCTTGCTCCTGCCGAGAAAGTATCC
ATCATGGCTGATGCAATGCGGCGGCTGCATACGCTTGATCCGGCTACCTGCCCATTCGACCACCAAGCGAAACATCGCATCGAGCGAGCACGTACTCGGATGGAAGC
CGGTCTTGTCGATCAGGATGATCTGGACGAAGAGCATCAGGGGCTCGCGCCAGCCGAACTGTTCGCCAGGCTCAAGGCGCGCATGCCCGACGGCGAGGATCTCGTCG
TGACACATGGCGATGCCTGCTTGCCGAATATCATGGTGGAAAATGGCCGCTTTTCTGGATTCATCGACTGTGGCCGGCTGGGTGTGGCGGACCGCTATCAGGACATA
GCGTTGGCTACCCGTGATATTGCTGAAGAGCTTGGCGGCGAATGGGCTGACCGCTTCCTCGTGCTTTACGGTATCGCCGCTCCCGATTCGCAGCGCATCGCCTTCTA
TCGCCTTCTTGACGAGTTCTTCTGATTAATTAACGATCGACAAGCTCTATTTTCTCCATAATAATGTGTGAGTAGTTCCCAGATAAGGGAATTAGGGTTCCTATAGG
GTTTCGCTCATGTGTTGAGCATATAAGAAACCCTTAGTATGTATTTGTATTTGTAAAATACTTCTATCAATAAAATTTCTAATTCCTAAAACCAAAATCCAGTACTA
AAATCCAGATCCCCCGAATTAATTCGGCGTTAATTCAGCATGGAGTCAAAGATTCAAATAGAGGACCTAACAGAACTCGCCGTAAAGACTGGCGAACAGTTCATACA
GAGTCTCTTACGACTCAATGACAAGAAGAAAATCTTCGTCAACATGGTGGAGCACGACACACTTGTCTACTCCAAAAATATCAAAGATACAGTCTCAGAAGACCAAA
GGGCAATTGAGACTTTTCAACAAAGGGTAATATCCGGAAACCTCCTCGGATTCCATTGCCCAGCTATCTGTCACTTTATTGTGAAGATAGTGGAAAAGGAAGGTGGC
TCCTACAAATGCCATCATTGCGATAAAGGAAAGGCCATCGTTGAAGATGCCTCTGCCGACAGTGGTCCCAAAGATGGACCCCCACCCACGAGGAGCATCGTGGAAAA
AGAAGACGTTCCAACCACGTCTTCAAAGCAAGTGGATTGATGTGATATCTCCACTGACGTAAGGGATGACGCACAATCCCACTATCCTTCGCAAGACCCTTCCTCTA
TATAAGGAAGTTCATTTCATTTGGAGAGAACACGGGGGACTCTTGACAGTAGTATAATATATTATCAAATTATTATGAACAGTACATCTATGTCTTCATTGGGAGTG
AGAAAAGGTTCATGGACTGATGAAGAAGATTTTCTTCTAAGAAAATGTATTGATAAGTATGGTGAAGGAAAATGGCATCTTGTTCCCATAAGAGCTGGTAACTATTA
AATTAACTATCACGTTATTTTTATTTGTCTTTCTGTCTCATTTTATTTGACGTTATTACGAATATCATCTGAAAATGTACGTGCAGGTCTGAATAGATGTCGGAAAA
GTTGTAGATTGAGGTGGCTGAATTATCTAAGGCCACATATCAAGAGAGGTGACTTTGAACAAGATGAAGTGGATCTCATTTTGAGGCTTCATAAGCTCTTAGGCAAC
AGGCATGCAAGTTTATGTTTTGACAAAATTTGATTAGTATATATTATATATACGTGTGACTATTTCATCTAAATGTTACGTTATTTTACGTAGATGGTCACTTATTG
CTGGTAGACTTCCCGGAAGGACAGCTAACGATGTGAAAAACTATTGGAACACTAATCTTCTAAGGAAGTTAAATACTACTAAAATTGTTCCTCGCGAAAAGATTAAC
AATAAGTGTGGAGAAATTAGTACTAAGATTGAAATTATAAAACCTCAACGACGCAAGTATTTCTCAAGCACAATGAAGAATGTTACAAACAATAATGTAATTTTGGA
CGAGGAGGAACATTGCA

Figure S3. Sequence of the donor template for targeted insertion into the *ANT1* locus. The 987 bp and 719 bp homology arms are highlighted in blue, the kanamycin resistance cassette in red and the 35S promoter in green.

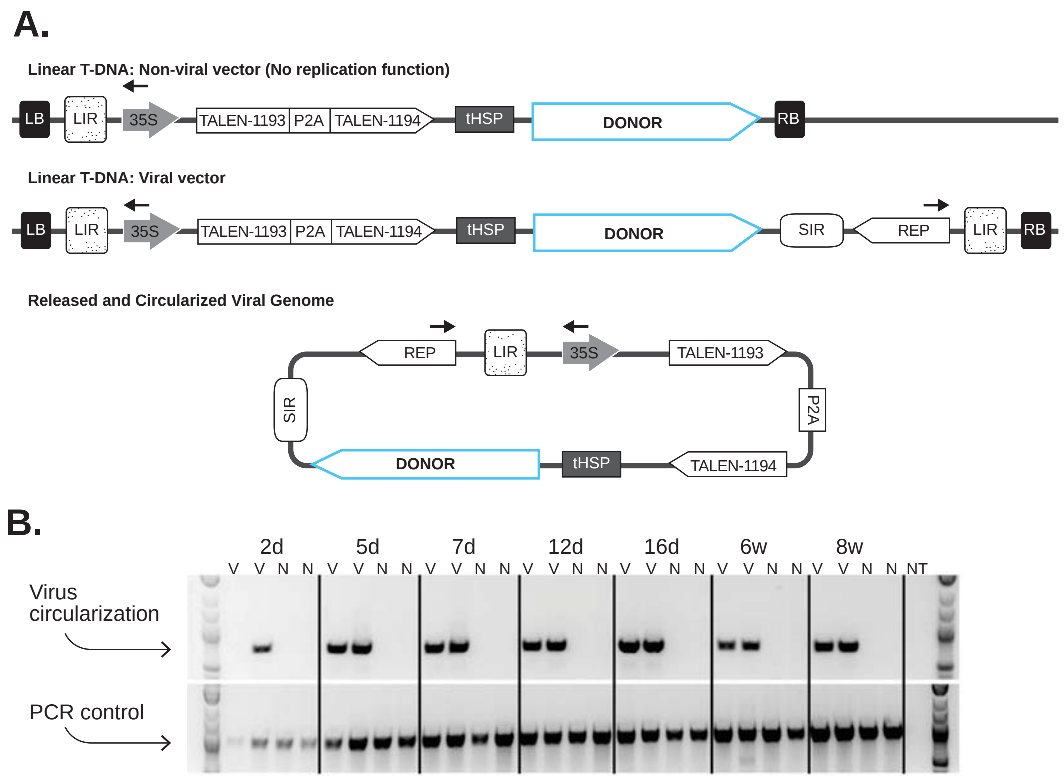

Figure S4.
PCR-based detection of circularized viral replicons in *Agrobacterium*-inoculated tomato explants. (A) Linear structures of the non-viral and viral GT vectors, representing the forms delivered to plant cells by *Agrobacterium*. Below the linear structures is shown the viral vector after release from the T-DNA and circularization. Primers used to detect circular replicons are indicat-ed by arrows. **(B)** PCR assays confirm presence of circular replicons from two days to eight weeks after Agrobacterium inocula-tion of cotyledonst transformed with the viral GT vector. No replicons were observed in cotyledons transformed with the non-viral GT vector. Two explants were tested for each vector at each time point. The PCR control used primers designed to amplify the *ANT1* gene. V, viral GT vector; N, non-viral GT vector; NT, no template control.

A.

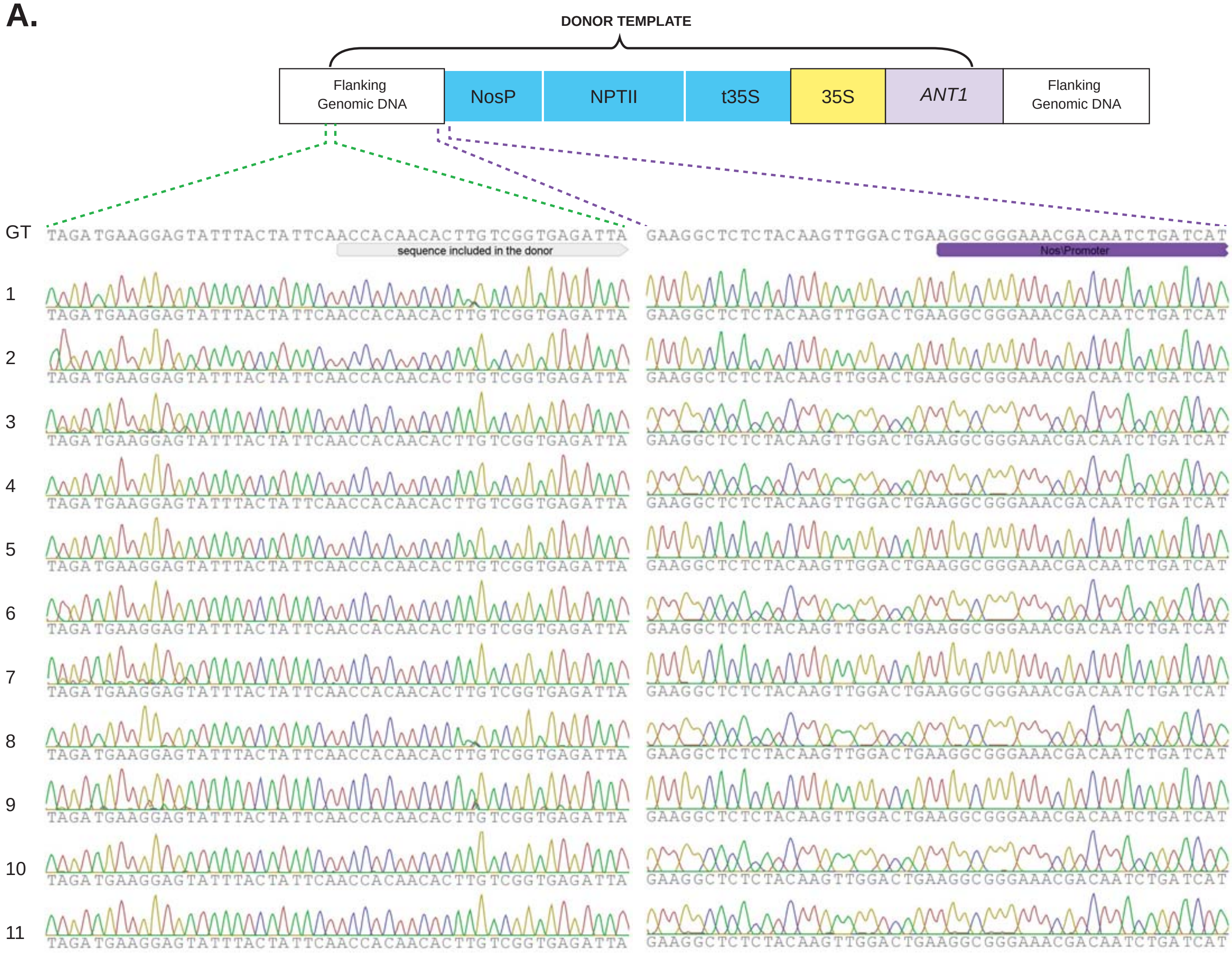

Figure S5. DNA sequences at the left recombination junction in 11 of 16 purple calli obtained from one transformation experiment. Sequences of a portion of the left homology arm and of the junction between the native *ANT1* locus and the insertion cassette are shown. All 11 sequences show a perfect match to the sequence expected for a precise HR-mediated targeted insertion event. No mutations were detected along the left homology arm sequence in any of the samples.

A.

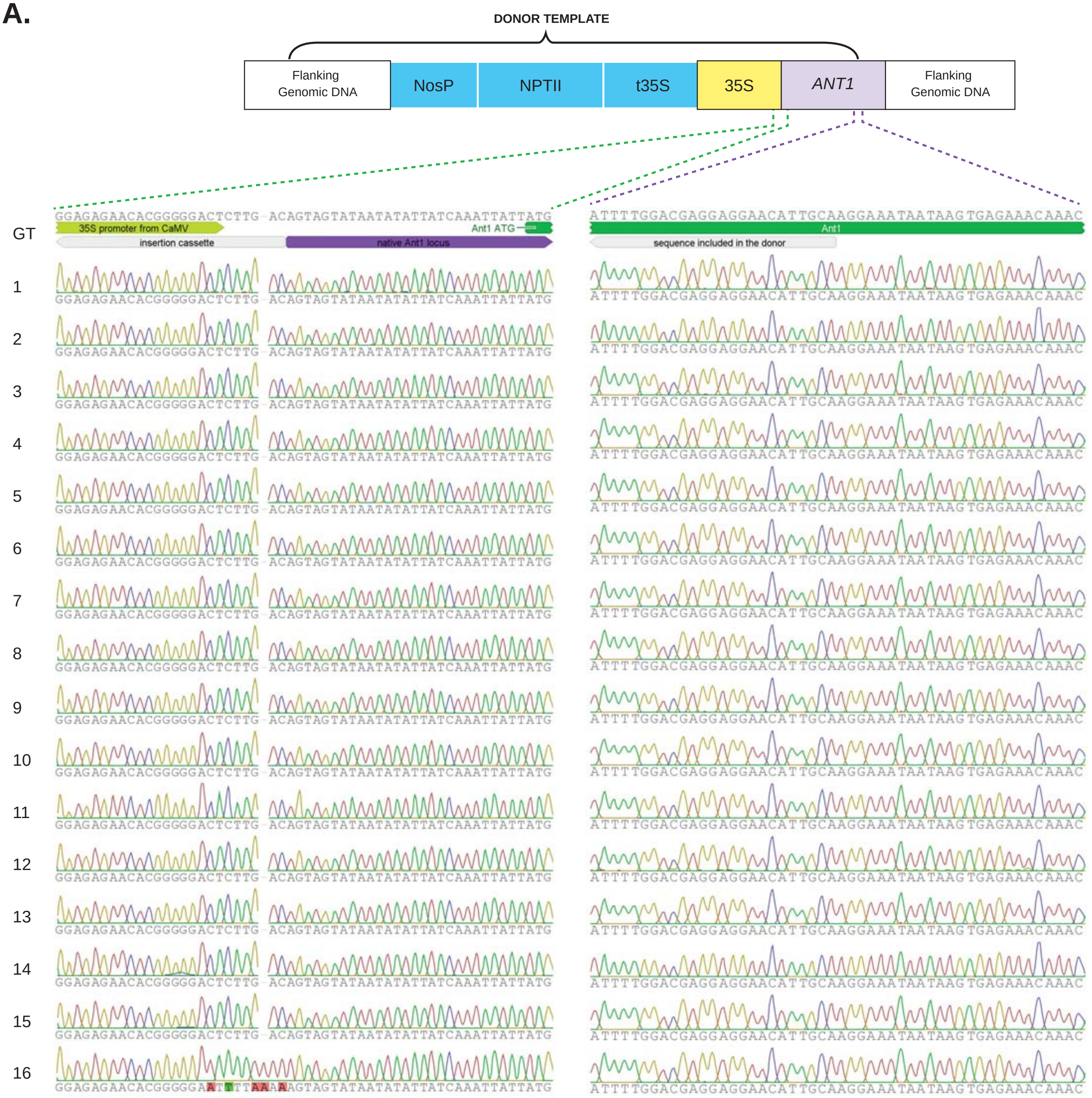

Figure S6. DNA sequences at the right recombination junction in 16 purple calli obtained from one transformation experiment. Sequences of a portion of the right homology arm and of the junction between the native *ANT1* locus and the insertion cassette are shown. Sequences of 15 samples show a perfect match to the sequence expected for a precise HR-mediated targeted insertion event. One sample had four single bp pair substitutions and a one bp insertion at the junction between the insertion cassette and the *ANT1* gene. No other mutations were detected along the right homology arm sequence in any of the samples.

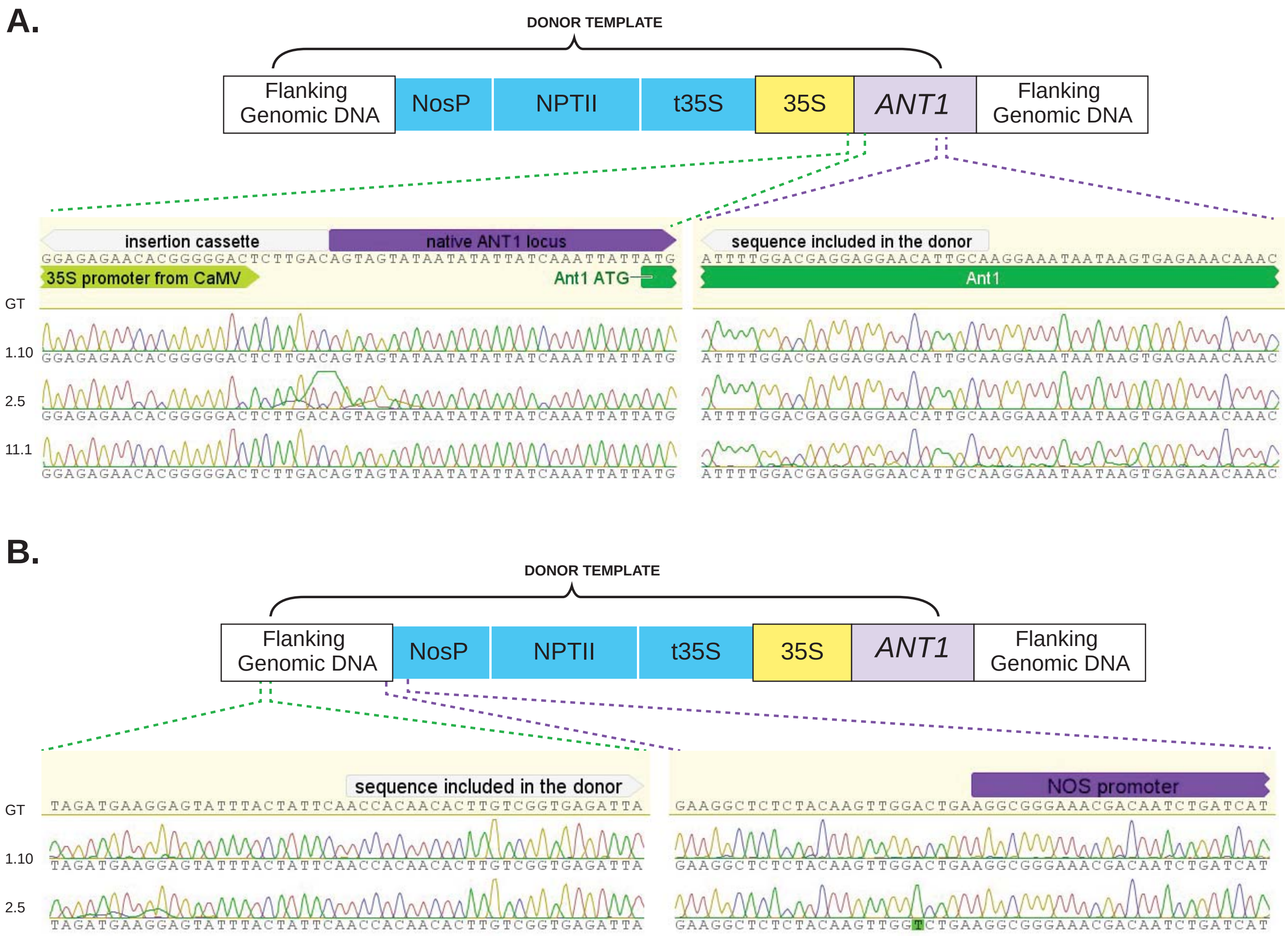

Figure S7. DNA sequence analysis of the recombination junctions in three selected purple plants – one each from event 1, 2 and 11. (A) Sequences of a portion of the right homology arm at the junction between the native *ANT1* locus and the insertion cassette are shown. Sequences of all 3 plants show a perfect match to the sequence expected for a precise HR-mediated targeted insertion event. No mutations were detected along the right homology arm sequence in any of the samples. **(B)** Sequences of a portion of the left homology arm at the junction between the native *ANT1* locus and the insertion cassette. Plant 2.5 carried a single nucleotide substitution at the border of the native *ANT1* locus and the insertion cassette, as well as another single nucleotide substitution within the NOS promoter in the insertion cassette (not shown). All other sequences showed a perfect match to the sequence expected for a precise HR-mediated targeted insertion event. Note that the left junction PCR reaction failed to produce a product in plant 11.1.

A.

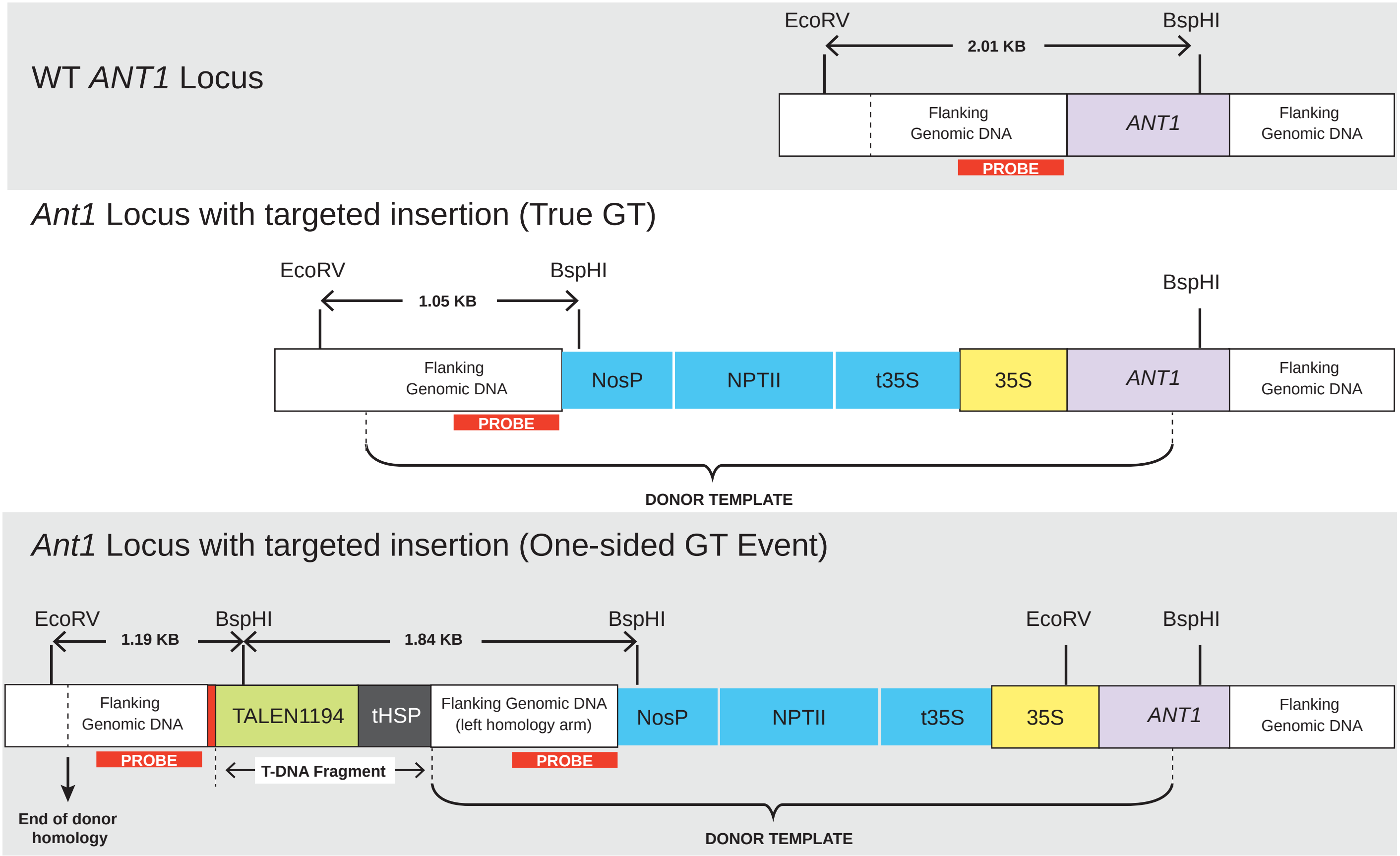

B.

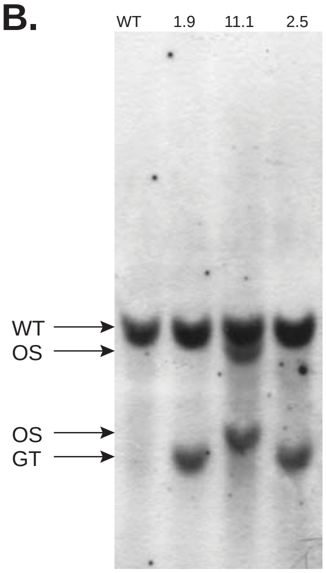

Figure S8. Southern blot analysis of BspHI + EcoRV digested genomic DNA from three selected purple plants (same as in Figure 4). (A) Diagrams of the wild type (WT) locus and loci that sustained a true GT event or a one-sided GT event. The probes are indicated as well as relevant restriction enzyme sites.
(B) The Southern blot is shown, and the band predicted for a true GT event is 1.05 kb. This band was found in plants 1.9 and 2.5. The bands predicted of a one-sided (OS) event are 1.84 kb and 1.19 kb and are observed in plant 11.1. All plants showed the 2.01 kb WT band. As in the Southern analysis performed with NsiI (Figure 4), no additional bands were detected, supporting the conclusion that the tested plants were free of random T-DNA integration.

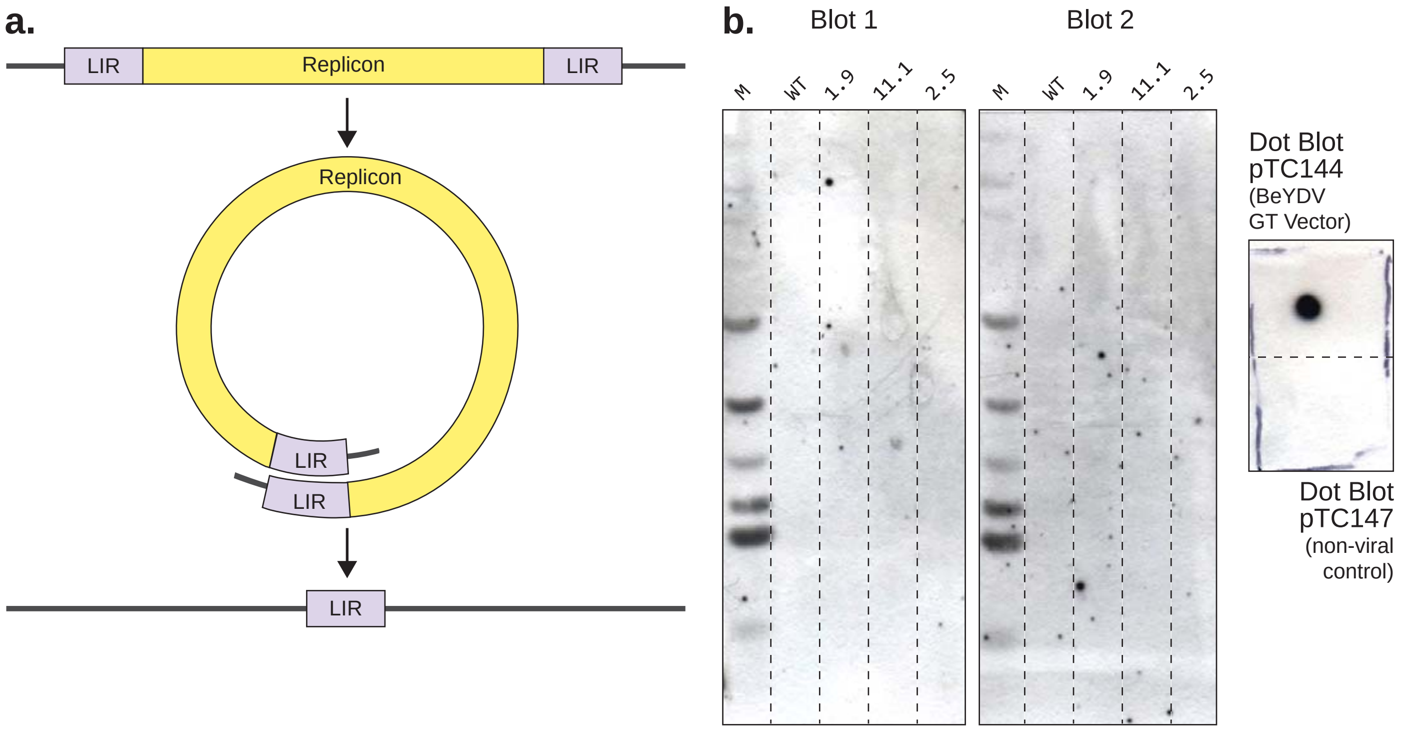

Figure S9. Detection of viral LIR sequences by Southern blot analysis using NsiI and BspHI + EcoRV digested genomic DNA from three selected purple plants. (A) Schematic of an intramolecular recombination event between the two LIR sequences that results in a T-DNA carrying a single LIR. (B) Two Southern blots are shown, hybridized with the LIR probe. No signals were detected in any of the samples, indicating the lack of LIR sequences in the genomes of these plants. Non-specific hybridization of the probe to the DNA ladder demonstrates that the DNA on the blot remained intact after the membranes were stripped and re-probed. The activity of the DNA probe was verified by hybridization of dot blots with two vectors with or without LIR sequences.

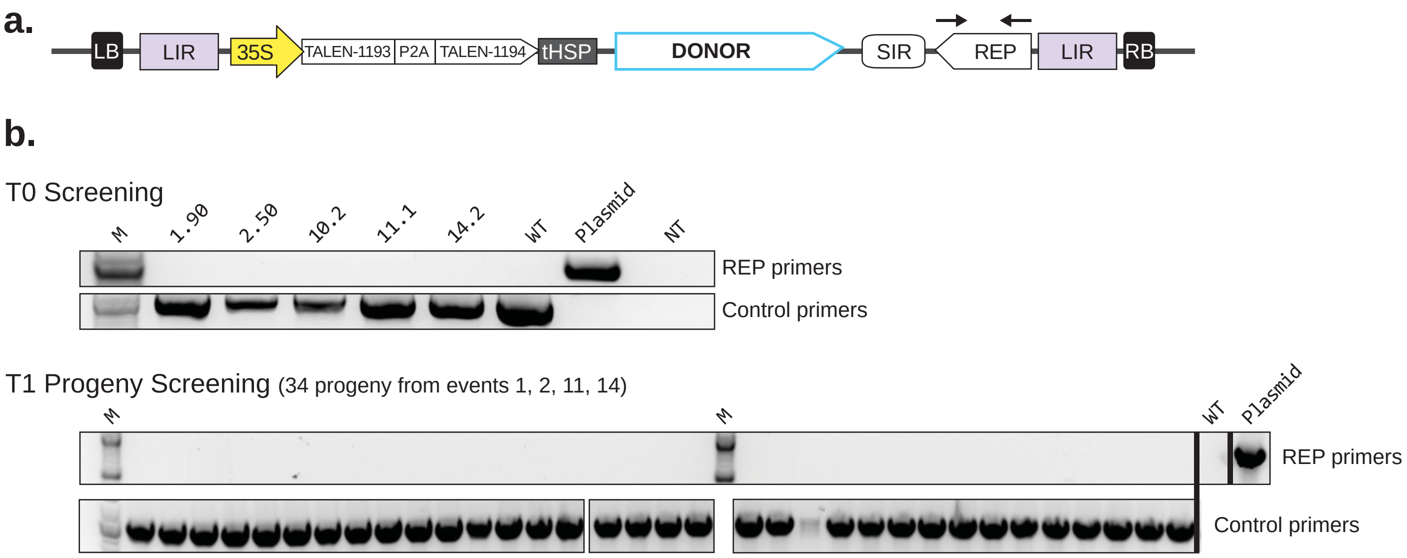

Figure S10. PCR analysis of random T-DNA integration. (A) Schematic of the T-DNA vector, pTC144. Primers used for T-DNA detection amplify REP sequence upstream of the right LIR and close to the right T-DNA border. This sequence is present in both linear T-DNA and circular replicons. (B) PCR screening of five T0 plants (one from each GT event) and 34 T1 progeny did not reveal the presence of T-DNA or replicons in any of the samples. Control primers (same as in Fig. 4) amplify *ANT1* sequence.

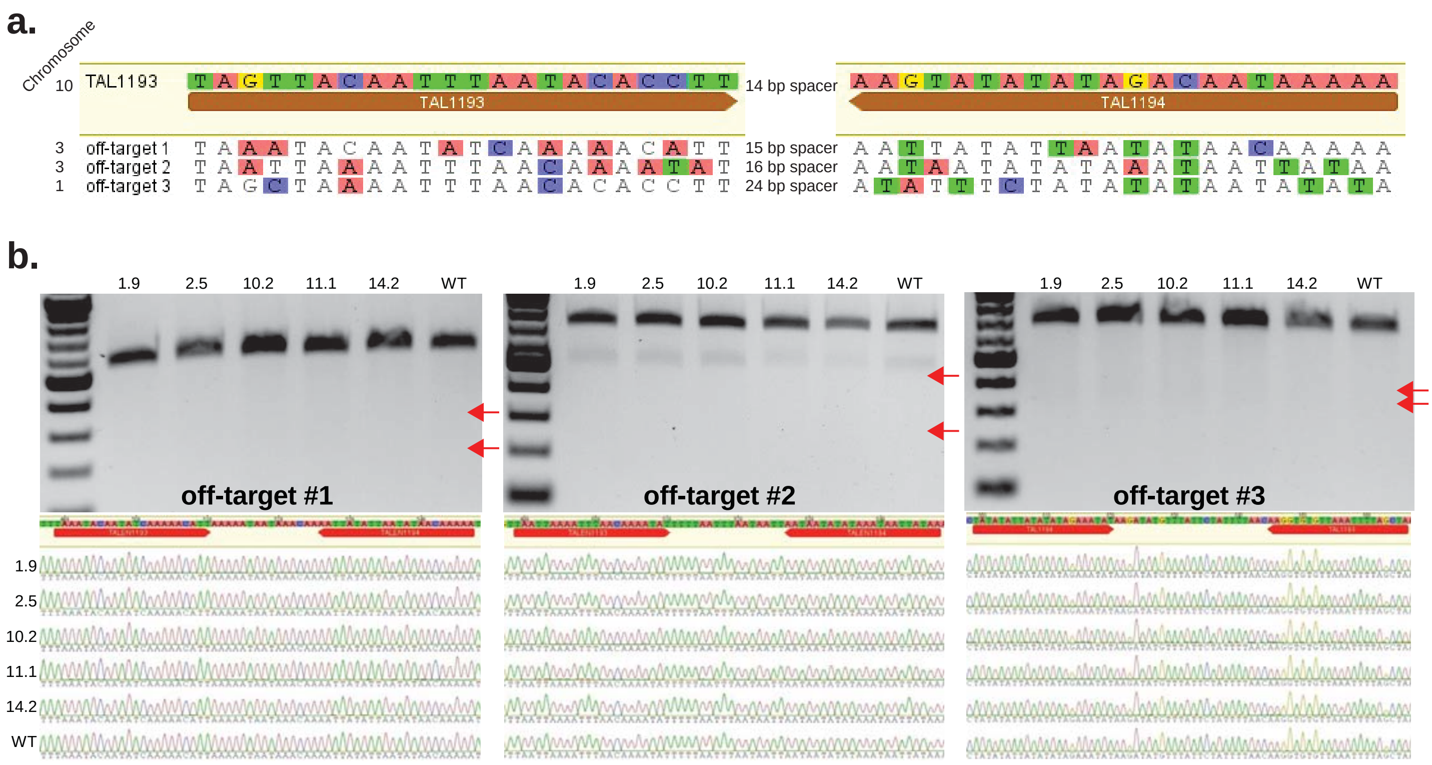

Figure S11. Analysis of three closest off-targets for TALEN1193/1194 in five independent GT events.
(A) Sequences of the three tested off-target sites. The T in position -1 is included. Mismatches with the on-target TALEN binding sites are highlighted. (B) T7EI assay and direct sequence analysis. Positions of expected cleavage products are marked by red arrowheads. Note that the cleavage product produced in all samples from off-target #2 is also present in the WT sample and therefore does not represent a TALEN induced indel. Sequence traces from PCR products for each off-target are shown. No double peaks were detected, suggesting that the sequences of both alleles of each off-target site are wild-type in all tested samples.

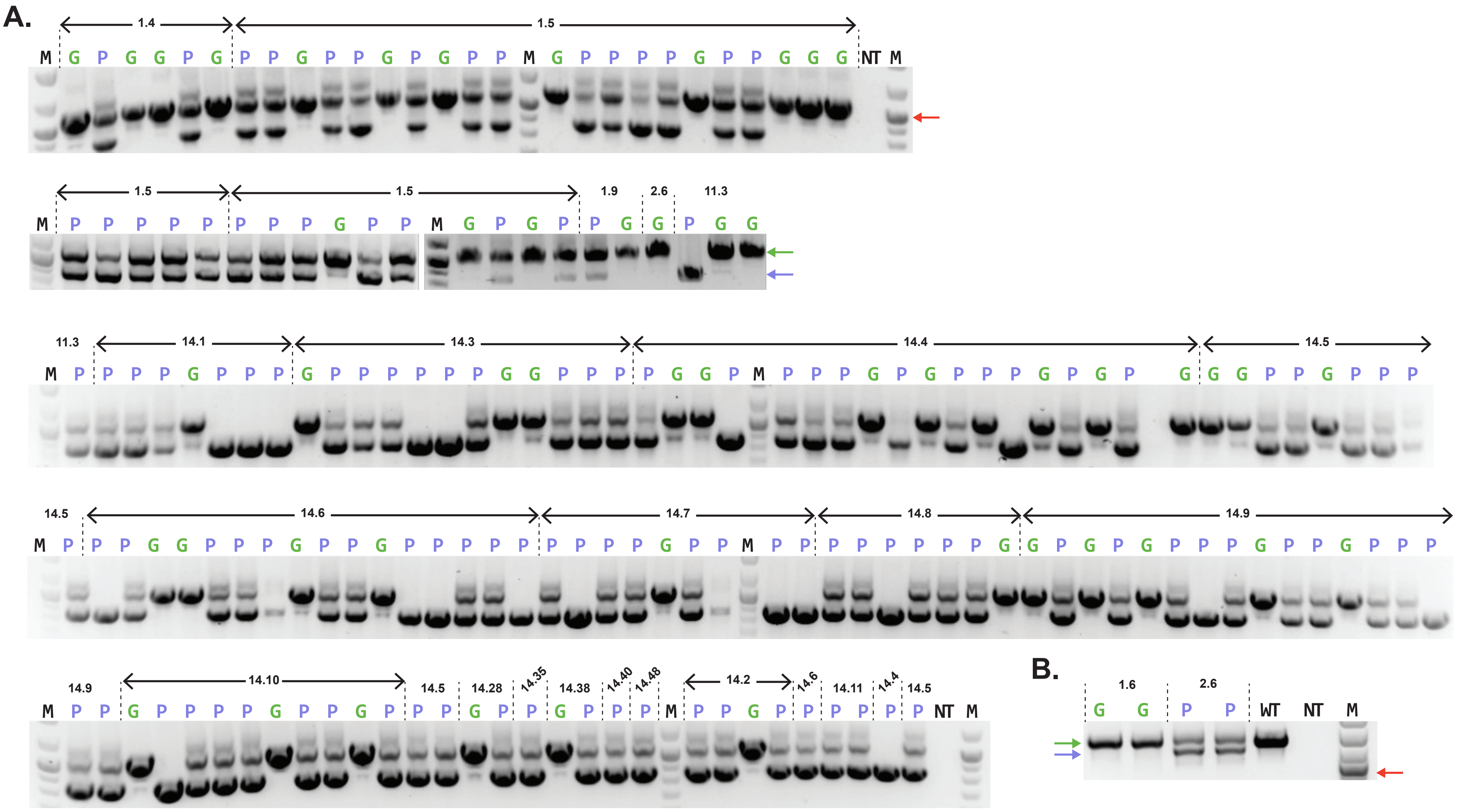

Figure S12. Multiplexed PCR analysis of 175 T1 progeny from four independent GT events (1, 2, 11 and 14). Primers TC097F, ZY010F and TC210R (Additional file 1: TableS1 - same as primers 1, 2 and 3 in Figure 6) were used in the same reaction. Green arrow marks the WT products, the purple arrow the GT products, and red arrow the 1.0 kb band in the DNA ladder. The phenotype of each seedling is marked by P (purple) or G (green). Parent plants are indicated with the number of the GT event and plant number separated by a period. Primer TC140R (Additional file 2: Table S1) was used instead of primer TC210R to screen the plants in **b**. Corresponding product sizes are 1491 bp for WT and 1280 bp for GT alleles. M: 2-Log DNA ladder (New England Biolabs). NT: no template control. WT: DNA from a non-transformed WT plant was used as control.

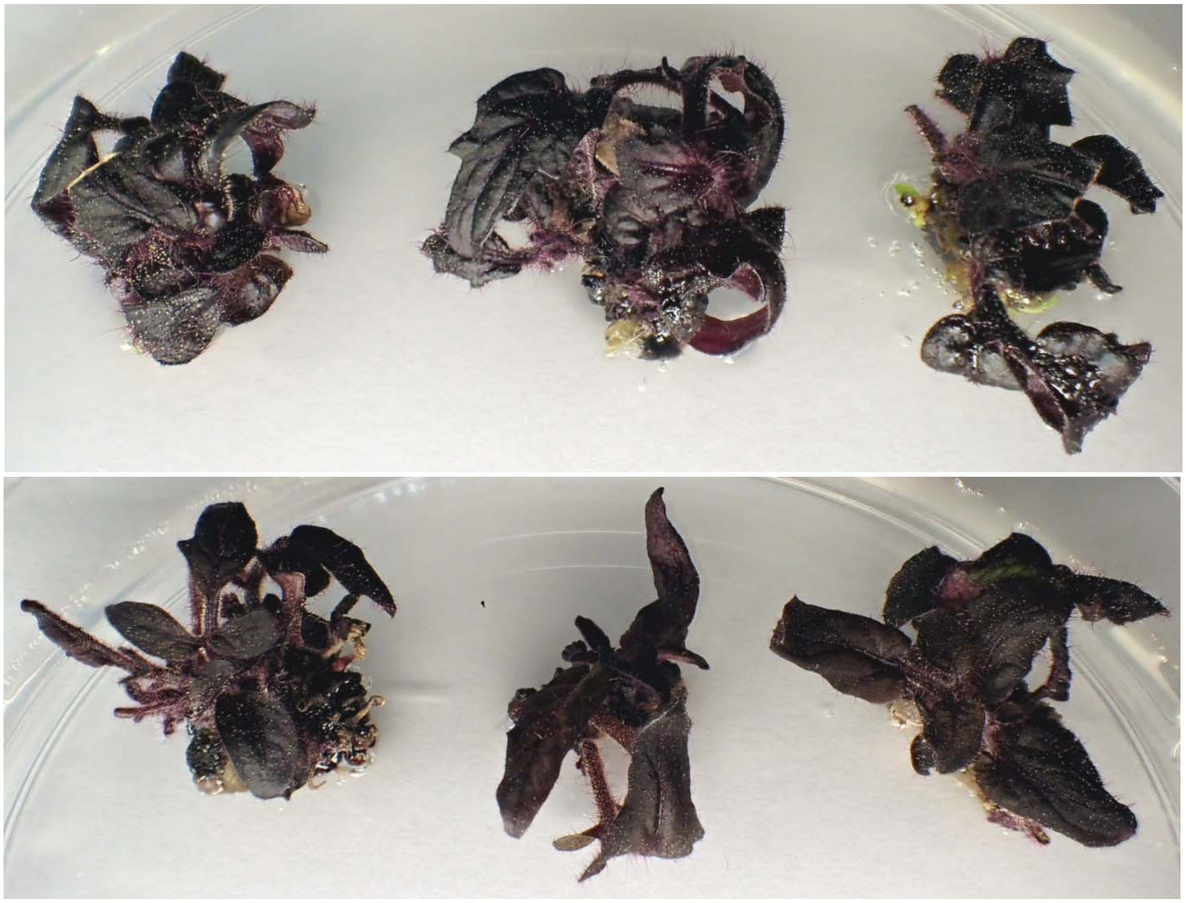

Homozygote

Heterozygote

Figure S13. Regenerative capacity of plants homo- and heterozygous for targeted promoter insertions. Homo- and heterozygote T1 seedlings were identified by PCR, and their cotyledons were regenerated on 2Z medium as described in the Methods section. Pictures were taken 12 weeks after inoculation with *Agrobacterium*. No significant difference in callus and shoot induction is visible.

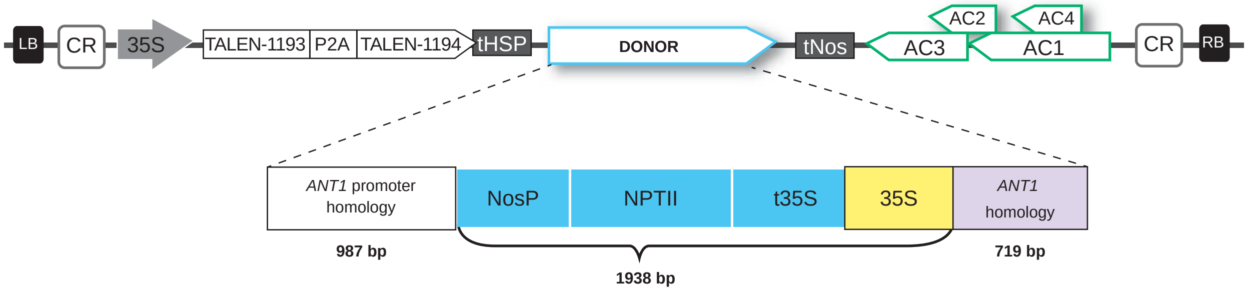

Figure S14. Structure of the ToLCV gene targeting T-DNA vector. LB, left T-DNA border; CR, ToLCV common region; 35S, cauliflower mosaic virus 35S promoter; tHSP, *Arabidopsis thaliana* heat shock protein 18.2 terminator; tNos, *Agrobacterium tumefaciens* nopaline synthase terminator; AC1, Rep gene; AC3, replication enhancer; AC2 and AC4, RNAi supressors; RB, right T-DNA border; NosP, *Agrobacterium tumefaciens* nopaline synthase promoter; NPTII, neomycin phosphotransferase gene for kanamycin resistance; t35S, CaMV 35S terminator.
