# Supplementary material for: High-frequency, precise modification of the tomato genome
Source: Genome Biol. 2015 Nov 6;16:232. doi: 10.1186/s13059-015-0796-9 (PMC4635538; doi:10.1186/s13059-015-0796-9)
Supplement: Additional file 2: Table S1. — List of primers used in the study. Table S2. List of constructs used in the study. (PDF 45 kb) [file 13059_2015_796_MOESM2_ESM.pdf]

Table S1. List of primers used in the study.

Primer Name	5'-3' Sequence	Application	Product Size (bp)
TC097F<br>TC097R	ACTTGATCTGTCATGTATGCTCA<br>AGATGCCATTTTCCTTCACCA	PCR-RE digestion assay to detect TALEN-induced mutation in protoplasts	452
ZY010F<br>TC140R	ACGTAAGGGATGACGCACA<br>TACCACCGGTCCATTCCCTA	Right GT junction detection	1280
TC144F<br>TC145R	CGGTAACAAAAGGCTACTCCGT<br>TTCAGTGACAACGTCGAGCA	Left GT junction detection	1709
TC140F<br>TC140R	GGAAAATGGCATCTTGTTCCC<br>TACCACCGGTCCATTCCCTA	ANT1 PCR control	1056
TC020F<br>TC210R	GAATGTATGATCACATTATAATGAATAAACAAATG<br>GTCCATCGATGCATCTGGAGT	One-sided event detection	3877
TC085F<br>ZY083R2	GCGAGGAGAAACCTTATGATC<br>CTGCGTAAGCCTCTCTAACCA	Virus circularization detection	780
TC100F<br>TC100R	GGGATCCCACCTTTTATTTTC<br>ACAACAACATGCCTTCTGCTAGTAAGAACTTC	T-DNA and replicon detection (REP)	957
TC080F<br>C2R	ACCACAACACTTGTCGGTGA<br>CGATTGGGAAACCCATCTTTC	Southern probe amplification (ANT1)	871
TC101F<br>TC246R	GCAGAAGGCATGTTGTTGTG<br>GAGGGTCGTACGAATAATTCG	Southern probe amplification (LIR)	363
NB492<br>TC126R	GCTACAGAGTTCTTGAAGTGGTG<br>GATAAGGAGCTCTACATTAAAAACGTCCGCAA	pTC130/131 Vector construction	1587
TC131F<br>TC131R	CGCGTAGTCCTCGGTAACCACAACACTTGTCGGTGA<br>CCTTCAGTCCAACTTGTAGAGAGCCTTC	ANT1 left homology for Gibson assembly of pTC144/146/206	1011
TC132F<br>TC132R	CAAGTTGGACTGAAGGCGGGAAACGAC<br>ATCCTAGGATCTCATTGCCCCCCGG	NOS promoter for Gibson assembly of pTC144/146/206	390
TC133F<br>TC133R	CAATGAGATCCTAGGATGGGGATTGAACA<br>GACTCCATGCTGAATTAACGCCGAATTAA	NPTII plus 35S polyA for Gibson assembly of pTC144/146/206	1049
TC134F<br>TC134R	TAATTCAGCATGGAGTCAAAGATTCAAA<br>GTCAAGAGTCCCCCGTGTTC	35S promoter for Gibson assembly of pTC144/146/206	552
TC127F<br>TC135R2	ACGGGGGACTCTTGACAGTAGTATAATATATTATCAAATTATTATGAA<br>TGACTTGAAGTACACTCATTTAAATGCAATGTTCCTCCTCGTCC	ANT1 right homology for Gibson assembly of pTC144/146/206	760
TC127F<br>TC079R	ACGGGGGACTCTTGACAGTAGTATAATATATTATCAAATTATTATGAA<br>GGGGAAATTCGAGCTGTTAATCAAGTAGATTCCATAAGTCA	ANT1 gene amplification for pTC147 construction	1071
TC09 7_ampli_F2<br>TC097_ampli_R	TCGTCGGCAGCGTCAGATGTGTATAAGAGACAGGAAATGAAAGATGGGTTTCCC<br>GTCTCGTGGGCTCGGAGATGTGTATAAGAGACAGAGATGCCATTTTCCTTCACCA	Deep sequencing analysis	340
ZY010F	ACGTAAGGGATGACGCACA	Multiplex PCR for T1 progeny genotyping	861 (GT)
TC097F<br>TC210R	ACTTGATCTGTCATGTATGCTCA<br>GTCCATCGATGCATCTGGAGT	Multiplex PCR for T1 progeny genotyping	1072 (WT)
TC331F<br>TC331R	ACTCCCCTCTGTCCCAATTT<br>CTCATCCAACAACCCCAGTC	Off-target 1	600
TC332F<br>TC332R	TTAGAGGTGTTCGTGGTTCG<br>GCCCACAAATCTCATAAATTCCC	Off-target 2	735
TC333F<br>TC333R	GATGCCATGTGTGTCTGAGA<br>TGTCCTTTGATCTTGTGGCC	Off-target 3	719

Table S2. List of constructs used in the study.

Construct Name	Application
**pTC144**	BeYDV ANT1-GT T-DNA vector with TALEN1193/1194 in the replicon
**pTC146**	BeYDV ANT1-GT T-DNA vector with TALEN1193/1194 outside the replicon
**pTC147**	35S:ANT1 expressing, non-replicating T-DNA transformation efficiency control
**pTC150**	BeYDV ANT1-GT T-DNA vector without the nuclease
**pTC151**	Non-replicating ANT1-GT T-DNA vector
**pTC206**	BeYDV ANT1-GT T-DNA vector without kanamycin selection
**pTC208**	ToLCV ANT1-GT T-DNA vector with TALEN1193/1194 in the replicon
**pTC217**	BeYDV ANT1-GT T-DNA vector with Cas9/gRNA1b in the replicon
**pTC223**	BeYDV ANT1-GT T-DNA vector with Cas9/gRNA7 in the replicon
